# Environmental pollution is associated with increased risk of psychiatric disorders in the US and Denmark

**DOI:** 10.1371/journal.pbio.3000353

**Published:** 2019-08-20

**Authors:** Atif Khan, Oleguer Plana-Ripoll, Sussie Antonsen, Jørgen Brandt, Camilla Geels, Hannah Landecker, Patrick F. Sullivan, Carsten Bøcker Pedersen, Andrey Rzhetsky

**Affiliations:** 1 Department of Medicine, Institute of Genomics and Systems Biology, University of Chicago, Chicago, Illinois, United States of America; 2 National Centre for Register-Based Research, Aarhus BSS, Department of Economics and Business Economics, Aarhus University, Aarhus, Denmark; 3 Centre for Integrated Register-based Research, CIRRAU, Aarhus University, Aarhus, Denmark; 4 The Lundbeck Foundation Initiative for Integrative Psychiatric Research, iPSYCH, Aarhus, Denmark; 5 Department of Environmental Science, Aarhus University, Roskilde, Denmark; 6 Department of Sociology and the Institute for Society and Genetics, University of California Los Angeles, Los Angeles, California, United States of America; 7 Department of Medical Epidemiology and Biostatistics, Karolinska Institutet, Stockholm, Sweden; 8 Departments of Genetics and Psychiatry, University of North Carolina, Chapel Hill, North Carolina, United States of America; 9 Big Data Centre for Environment and Health, Aarhus University, Aarhus, Denmark; 10 Department of Human Genetics, Center for Data and Computing, University of Chicago, Chicago, Illinois, United States of America; Stanford University School of Medicine, UNITED STATES

## Abstract

The search for the genetic factors underlying complex neuropsychiatric disorders has proceeded apace in the past decade. Despite some advances in identifying genetic variants associated with psychiatric disorders, most variants have small individual contributions to risk. By contrast, disease risk increase appears to be less subtle for disease-predisposing environmental insults. In this study, we sought to identify associations between environmental pollution and risk of neuropsychiatric disorders. We present exploratory analyses of 2 independent, very large datasets: 151 million unique individuals, represented in a United States insurance claims dataset, and 1.4 million unique individuals documented in Danish national treatment registers. Environmental Protection Agency (EPA) county-level environmental quality indices (EQIs) in the US and individual-level exposure to air pollution in Denmark were used to assess the association between pollution exposure and the risk of neuropsychiatric disorders. These results show that air pollution is significantly associated with increased risk of psychiatric disorders. We hypothesize that pollutants affect the human brain via neuroinflammatory pathways that have also been shown to cause depression-like phenotypes in animal studies.

## Abstract 2

Exploratory analyses of two independent datasets comprising 151 million Americans and 1.4 million Danes revealed that polluted air was associated with a significantly increased risk of psychiatric disorders, including bipolar disorder and schizophrenia.

**Editor’s Note**

This Short Report received both positive and negative reviews by experts. The Academic Editor has written an accompanying Primer that is published alongside this article (https://doi.org/10.1371/journal.pbio.3000370). The linked Primer presents a complementary expert perspective; it discusses how the current study should be interpreted, given the modest effect size, potential biases, and other limitations.

## Introduction

The increasing prevalence of mental disorders is a major global problem that affects millions of people every year. In addition to personal suffering, psychiatric disorders are associated with significant societal costs [1]. A number of putative contributors to the etiology of these illnesses have been identified, but the majority of risk factors remain unknown. Mental illnesses such as bipolar disorder and schizophrenia develop due to a complex interplay of genetic predispositions and life experiences or exposures [2–5]. In the last decade, the genetic underpinnings of mental disorders have been extensively studied. For instance, recent work has identified 145 genome-wide significant associations for schizophrenia [6, 7]. However, genetics alone cannot account for full phenotypic variation in mental health and disease, and it has long been believed that genetic, neurochemical, and environmental factors interact at many different levels to play a role in the onset, severity, and progression of these illnesses. The major neuropsychiatric disorders cover a broad range of heritability values, leaving ample room for environmental influences to play a role. From a comprehensive twin meta-analysis [8], environmental effects contribute to a 55% to 66% risk for major depression, 32% risk for bipolar disorder, and 23% risk for schizophrenia. Increased knowledge of environmental risk factors is therefore vital for a more comprehensive understanding of disease causation.

What aspects of human environments are driving psychiatric and neurological disease prevalence? Recent umbrella reviews of epidemiological studies analyzing putative risk factors associated with common psychiatric and neurological disorders suggest several contributing factors to mental health and well-being, such as individual attributes and behavior (medical illness, stressful life events, substance abuse, cognitive and/or emotional immaturity), social circumstances (poor access to basic services, unemployment, poverty, neglect, social injustice, relationship conflicts, work stress, exposure to violence, and abuse), and environmental factors (occupational exposure, and exposure to pollution) [9–11]. These reviews stressed that well-designed and adequately powered studies are necessary to map the environmental risk factors for psychiatric disorder. Studies of gene-environment interactions in the context of psychiatric disorders likewise point to a wide range of factors interacting with genotype in mental disorder prevalence [2, 12–15]. Historically, most of the attention to the environment as a causal factor in these studies has focused on home or family environments, with an empirically-justified emphasis on childhood adversity and trauma, [16] and, more recently, on prenatal influences [17].

Far fewer studies have explored the links between physical environments and mental illnesses (see [18–22]), with a small subset of these specifically focused on environmental pollution or its constituent toxicants [23]. Yet concern has been growing about the diverse negative health effects of air pollution, raising the possibility that air quality may play an important role in mental health and cognitive function. While the study of air pollution and health was originally driven by dramatic events and drastic outcomes such as mortality during 1930 Meuse Valley fog [24] due to the combination of industrial air pollution and climatic conditions, and the 1952 Great London Fog event [25–27], in which a multiple day temperature inversion concentrated coal-based air pollutants and resulted in thousands of deaths, attention has been turning to the question of chronic exposures and chronic diseases, including neurodevelopmental and neurodegenerative conditions [28, 29]. More recent events, such as the Eastern China smog in 2013 [30] and the New Delhi smog in 2017 [31] saw air pollution measurements reach record levels, conditions that led to significant increases in morbidity and mortality rates. Such events have led to considerable debate, along with an upsurge of environmental research, new government regulation (e.g., the Clean Air Act of 1956 in the UK and the Chinese Air Pollution Control Law in 2015), and heightened public awareness of the relationship between air quality and health. Increasing interest in the effect of pollution on neuropsychiatric disorders has only recently begun to direct attention toward the brain, with in vitro and animal model studies lending mechanistic insight into how air pollution components can be neurotoxic [32, 33].

Our exploratory analysis and conclusions concerning the significant associations between environmental quality and the rates of neuropsychiatric disorders are based on 2 independent, very large datasets. The first dataset is the IBM Health MarketScan Commercial Claims and Encounters Database [34], comprising insurance claims for 151,104,811 unique US individuals from 2003–2013. MarketScan was previously used for numerous studies involving, for example, estimation of prevalence of diseases in the US: traumatic brain injury [35, 36], attention deficit-hyperactivity disorder (ADHD) [37], epilepsy [38, 39], and depression [40]. The second dataset is the collection of Danish national treatment and pollution registers [41] comprising all individuals born in Denmark between January 1, 1979, and December 31, 2002, who were alive and residing in Denmark at their 10th birthday (1,436,702 unique individuals).

The environment for the US part of this study appears as 3 sets of variables at the county level: (i) quality of air, water, land, and “built” environment (e.g., amount of vehicular traffic, transit access, and pedestrian safety); (ii) weather indices split into number of days with at least 4 hours of pleasant weather (defined according to the design standards for climate-controlled buildings) and number of days with at least 4 hours of harsh (either too hot or too cold) weather (this last group of factors is useful in dissecting the outdoor environment’s positive [open-air activities] and negative [pollution] influences); and (iii) sociodemographic factors, such as median income, population density, and urbanicity, which are known risk factors for many psychiatric disorders. Therefore, individuals’ exposures to pollutants were measured at a county level for the US data. For the Denmark counterpart of our analysis, the environmental factors were estimated as exposure to air pollution during the initial 10 years of life. Our hypothesis was that these environmental factors causally contribute toward the onset and development of the psychiatric disorders in exposed individuals.

## Results

For the US cohort, we studied 4 psychiatric and 2 neurological conditions: bipolar disorder, major depression, personality disorder, schizophrenia, epilepsy, and Parkinson disease, each defined by sets of specific International Classification of Diseases, Ninth Revision, Clinical Modification (ICD-9-CM) codes (see Methods section). When we refer to these 6 conditions below, we are explicitly referring to data captured by IBM MarketScan database, which is the treated prevalence inferred from US insurance claims (see Table 1); because the data were potentially influenced by reporting biases, we refer to the IBM MarketScan disease rates as raw rates, to be further adjusted for confounders.

**Table 1:** Demographics of unique persons in IBM MarketScan database with at least one health insurance claim with diagnosis of bipolar disorder, schizophrenia, Parkinson disease, personality disorder, epilepsy, or major depression during 2003 to 2013.

	Category	Total (% of total)	Bipolar disorder	Schizophrenia	Parkinson disease	Personality disorder	Epilepsy	Major depression
**Public sources (see S1 Table)**	**Prevalence**	US population	2.8% (NIMH, 18+)	0.5%–1.0% (NIMH)	0.1%–0.2% (NIH)	1.4% (NIMH)	1.2% (CDC)	6.7% (NIMH, 18+)
**IBM MarketScan**	**Unique cases**	151,104,811 (100%)	1,238,255 (0.82%)	831,426 (0.55%)	243,493 (0.16%)	234,341 (0.15%)	937,933 (0.62%)	10,041,023 (6.64%)
**Sex**	**Male**	73,565,201 (48.69%)	476,315 (38.47%)	379,449 (45.64%)	137,854 (56.61%)	91,464 (39.03%)	433,571 (46.23%)	3,224,124 (32.1%)
	**Female**	77,539,610 (51.31%)	761,940 (61.53%)	451,977 (54.36%)	105,639 (43.39%)	142,877 (60.97%)	504,362 (53.77%)	6,816,899 (67.9%)
**Age**	**0–5**	13,596,589 (9%)	6,607 (0.53%)	3,806 (0.46%)	251 (0.1%)	3,967 (1.69%)	70,296 (7.49%)	31,135 (0.31%)
	**6–17**	24,381,821 (16.4%)	174,027 (14.05%)	74,494 (8.96%)	586 (0.24%)	38,542 (16.45%)	149,807 (15.97)	907,981 (9.04%)
	**18–30**	30,976,117 (20.5%)	276,735 (22.35%)	92,948 (11.18%)	1,342 (0.55%)	53,858 (22.98%)	148,636 (15.85%)	1,747,820 (17.41%)
	**31–40**	23,030,236 (15.24%)	241,809 (19.53%)	70,647 (8.5%)	2,890 (1.19%)	41,575 (17.74%)	118,967 (12.68%)	1,913,715 (19.06%)
	**41–50**	23,563,254 (15.59%)	248,854 (20.1%)	104,391 (12.56%)	10,586 (4.35%)	42,987 (18.34%)	143,477 (15.3%)	2,195,617 (21.87%)
	**51–65**	27,259,464 (18.04%)	238,454 (19.26%)	187,510 (22.5%)	63,903 (26.24%)	39,884 (17.02%)	197,255 (21.03%)	2,478,835 (24.69%)
	**65 plus**	8,297,330 (5.49%)	51,769 (4.18%)	297,630 (35.8%)	163,935 (67.33%)	13,528 (5.77%)	109,495 (11.67%)	765,920 (7.63%)
**Subjects mappable to US counties in IBM MarketScan**	**Unique cases**	100,316,345 (66.4%)	918,065 (74.1%)	422,654 (50.8%)	63,575 (26.1%)	170,693 (72.8%)	633,954 (67.6%)	7,170,269 (71.4%)

**Abbreviations:** CDC, Centers for Disease Control and Prevention; NIH, National Institutes of Health; NIMH, National Institute of Mental Health

### Spatial patterns of putative environmental risk factors in the US

**Fig 1:**
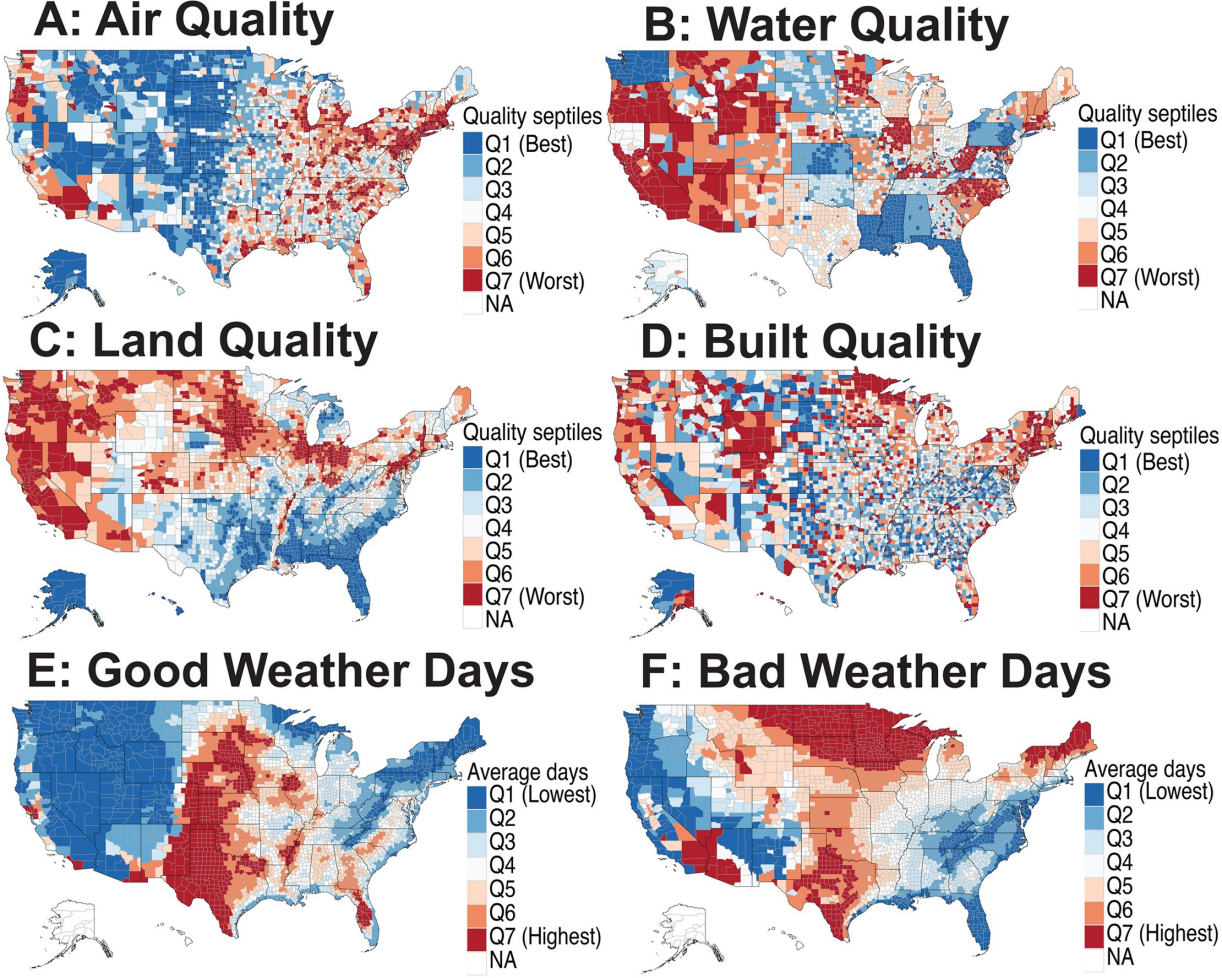
Spatial patterns of putative environmental risk factors in the US. The county-level environmental quality is assessed by the EQIs designed by the US EPA. (A) A map showing the EPA air quality index across US counties divided into septiles such that Q1 represents the best and Q7 represents the worst air quality regions. The EPA designed the index based on the measurements of 87 pollutants. (B) A map showing the EPA water quality index across counties constructed by the analysis of 80 water quality indicator variables. (C) EPA land quality index map constructed by the analysis of 26 land quality indicator variables. (D) EPA built quality index designed by the analysis of 14 built quality indicators (e.g., amount of vehicular traffic, transit access, and pedestrian safety). (E) A county-level map showing the average number of good weather days that indicated whether at least 4 hours in a diurnal cycle were in a “comfort zone,” defined as a 4-point patch with vertices in temperature and humidity space (temperature [18°C, 27°C, 27 °C, 18 °C] and relative humidity [6.71%, 8.85%, 13.85%, 10%]). (F) A county-level map showing average number of bad weather days that indicated whether at least 4 hours in a diurnal cycle were in an “extremely uncomfortable zone,” defined as <−5 °C or >35 °C. For both the “good weather days” and “bad weather days,” the number per year was averaged over the years during the period 2003–2012. The underlying data for producing these maps can be found in S1 Data. EPA, Environmental Protection Agency; EQI, Environmental Quality Index; NA, not available.

Spatial distribution of environmental risk factors varies significantly across the US (see Fig 1A–1D). Air quality (Fig 1A) is predictably worse near larger cities on both the US East and West Coasts while generally much better in the middle of the country. Water quality (Fig 1B) measurements showed very little variation across the US and is generally worse in the western US, as well as in some interior states (e.g., Wyoming and Illinois). Resolution of the water quality data facet is not very high, as county water quality descriptors closely follow state boundaries. Land quality (Fig 1C) appears to be worse in the north of the continental US as well as in the west. Importantly, land quality is not highly correlated with air quality across geographical space, facilitating the disentanglement of associations between factors. Built quality (Fig 1D) is patchy rather than continuous across counties. Regarding fair- and poor-weather days (Fig 1E and Fig 1F), central US counties far from coasts tend to have many poor-weather days, whereas coastal areas tend to be enriched with fair-weather days. Continental counties are correlated with a higher number of poor- and fair-weather days. The sociodemographic factors, including population density, urbanicity, insurance status, and poverty, showed variable patterns across the US (S1 Fig).

### Raw prevalence, sex ratio, and spatial disease patterns in the US

**Fig 2:**
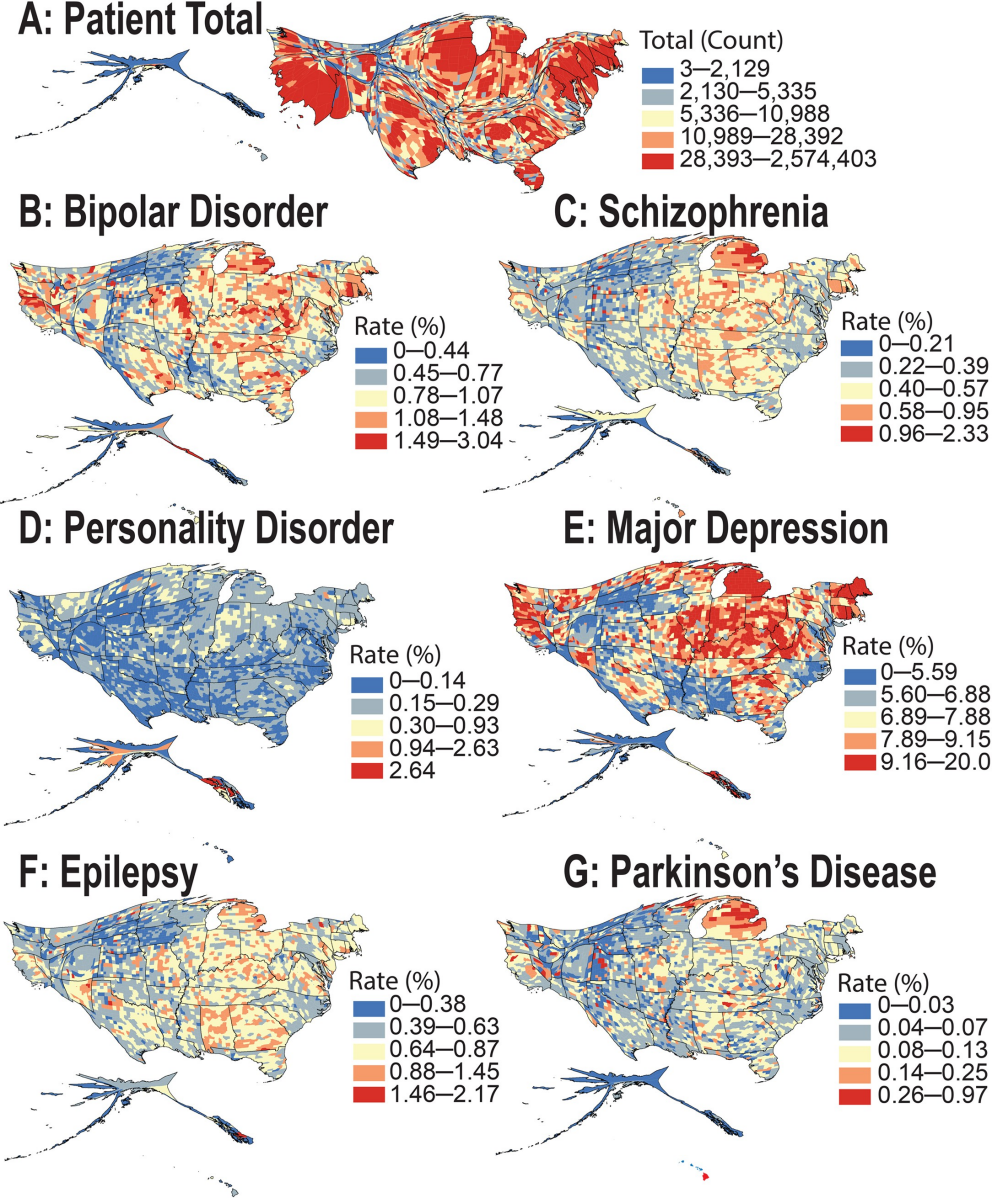
Cartogram maps showing the spatial patterns of apparent neurological and psychiatric disorder prevalence inferred from IBM MarketScan database. (A) Cartogram map of the total patient population (151 million) present in the MarketScan database during 2003–2013. County and state land areas are rescaled in proportion to their patient population, producing distorted maps. The squeezed regions contribute smaller shares of patient population compared to their corresponding land area and vice versa. The total map area remains the same. The subsequent cartogram maps show the prevalence of 4 psychiatric disorders: (B) bipolar disorder, (C) schizophrenia, (D) personality disorder, and (E) major depression, and 2 neurological disorders: (F) epilepsy and (G) Parkinson disease. The underlying data for producing these cartogram maps can be found in S1 Data.

From the health insurance claims analysis of over 151 million individuals represented in the IBM MarketScan database (during 2003–2013), the observed spatial patterns for the raw prevalence of 4 psychiatric and 2 neurological disorders in the US differ geographically to a remarkable extent (Fig 2A–2G, S2 Fig and S3 Fig). The raw (unadjusted) prevalence rates for bipolar and personality disorders were 0.82% and 0.15%, respectively, with both disorders 1.6 times more prevalent among female patients. The prevalence of major depression was 6.64% and was 2.1 times more common among women. Prevalence of schizophrenia and epilepsy was 0.55% and 0.62%, respectively, with both disorders at 1.2 times higher prevalence among female patients. In contrast, Parkinson disease was 1.3 times more common in males, with an overall prevalence of 0.16% (see Table 1). Note that after correcting for potential confounders [regression analysis], we found that the adjusted rates of bipolar disorder and personality disorder were 1.5 times higher among women. The rate of major depression was twice as high—and the rate of epilepsy was 1.12 times higher—among female patients. There was no significant difference in the adjusted rate of schizophrenia in male and female populations. These MarketScan prevalence estimates are in excellent agreement with those published previously (S1 Table).

Areas of the country distant from large bodies of water (in the continental US) are the most enriched for neuropsychiatric disorders across the board. This is particularly evident for major depression and bipolar disorder, and in Kentucky and Missouri, when comparing Fig 2A to the rest of the subfigures. At the state level, Alaska shows more psychiatric disorder diagnoses than expected for the overall population size—particularly for personality disorders and schizophrenia. Hawaii shows higher-than-expected rates of Parkinson disease and schizophrenia, whereas Michigan has an apparent increased prevalence of Parkinson disease, major depression, bipolar disorder, and schizophrenia. Our mixed-effect regression analyses suggested that Michigan’s apparent higher rate across all disorders is associated with reporting biases, visible in our analysis as high, state-specific random effects. The US East Coast experiences a higher prevalence of these phenotypes than the West Coast (S2 Fig and S3 Fig). Geospatial clusters with a high prevalence of major depression are observed among almost all counties of Michigan, New Hampshire, and Maine (Fig 2E and S3 Fig).

### Association between environmental factors and the risk of neurological and psychiatric disorders in the US

We considered several environmental factors for the prediction of neurological and psychiatric disease diagnosis among different age and sex groups at the US county level. These factors included the quality of air, water, land, built environment, and weather conditions. In addition, population density, median income, ethnic and racial composition, and the percentages of poor and insured populations were also included in the model. All environmental predictors were transformed into septiles, with Q1 representing the best-quality and Q7 representing the worst-quality regions (US counties). Similarly, for weather variables and sociodemographic covariates, Q1 and Q7 represent the regions with the least and highest percentages, respectively. We report the comparison of disease rates between referent group Q1 with all higher septiles (Q2–Q7).

**Fig 3:**
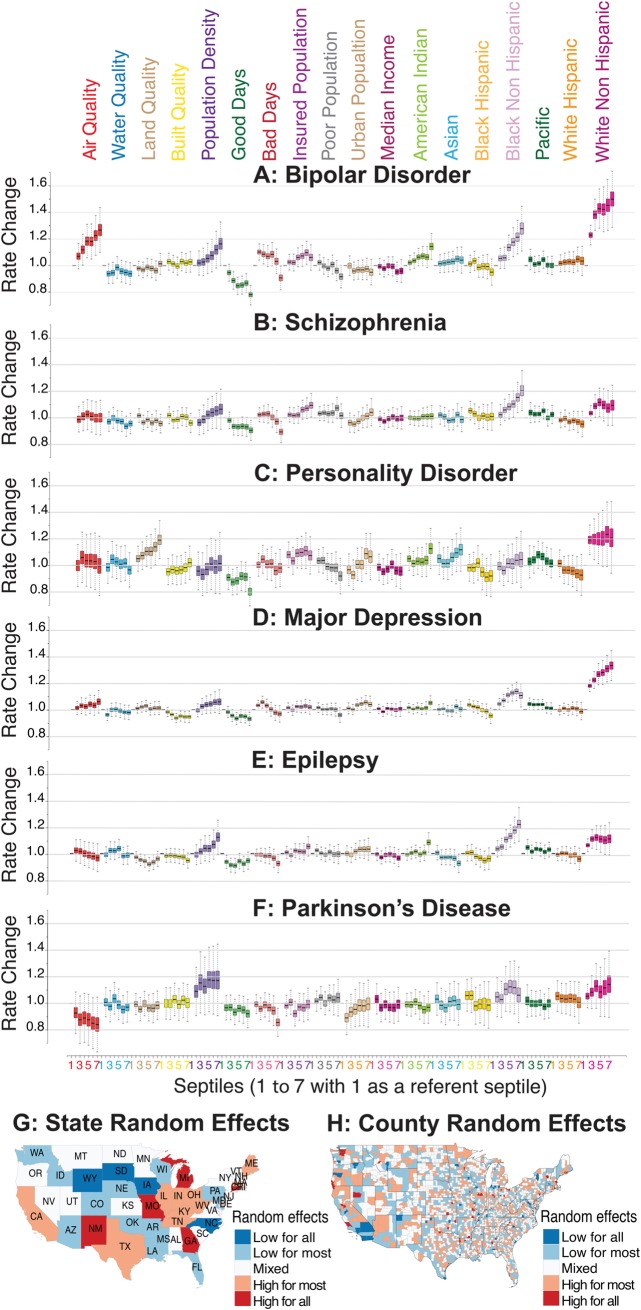
Relationship between environmental factors and neurological and psychiatric disorders in US. The results from the US data analysis in which all predictor variables are divided into septiles (7 groups) with each septile representing approximately 400 counties. Septile 1 (counties with the least exposure or the least percentage) is used as a referent to compare the disorder rates in the higher septiles (counties with systematically higher exposures or percentages). For air, water, land, and built qualities, a higher septile corresponds to the group of counties with poor quality. Similarly, for all other variables, a higher septile represents a higher fraction or the corresponding percentages. The estimated disorder rate from the mixed-effects regression model is shown for (A) bipolar disorder, (B) schizophrenia, (C) personality disorder, (D) major depression, (E) epilepsy, and (F) Parkinson disease. (G) Map showing the aggregated state-level random effects. The random effects for the 6 disorders are aggregated to produce 1 representative map. States shaded red show higher disorder diagnoses, and those shaded blue show lower disorder diagnoses that is not captured by our model. An apparent high neurological and psychiatric disorder rate in the states of Michigan, Missouri, Georgia, and New Mexico, and the apparent low rate in the states of South Dakota, Iowa, Wyoming, and North Carolina could be associated with reporting biases. (H) Map showing aggregated, county-level random effects. Random effects for the 6 disorders are aggregated to produce 1 representative map. Counties in red show higher disorder rates, and those in blue show lower disorder rates not captured by our model. County-level random effects can be thought of as residual variations not explained by fixed-effect predictors and state-level random effects. There are relatively few counties in which the county-level random effect is consistently low. For example, several counties are consistently low (San Diego, Imperial, Orange, and San Bernardino Counties in Southern California), and several counties are consistently high (San Luis Obispo County in California and Snohomish and King Counties in Washington). The underlying data for this figure can be found in S2 Table.

Reviewing results of the application of our mixed-effect Poisson regression model, we noticed significant variability in the prevalence of neuropsychiatric disorders across different racial/ethnic groups (Fig 3A–3F). The strongest predictor of mood disorders (major depression and bipolar disorder) in a county was its percentage of white individuals (using US Census race/ethnicity categories). By contrast, a higher percentage of black non-Hispanic individuals was associated with higher rates of schizophrenia and epilepsy.

The strongest predictor for bipolar disorder diagnosis, after a population’s ethnicity composition, was air quality (defined by the US Environmental Protection Agency [EPA] Environmental Quality Index [EQI]). The worst air quality was associated with an approximately 27% increase (95% credible interval [CrI] 15%–40%, *p_MCMC_*<10^−4^) in the apparent rate of bipolar disorder (Fig 3A and S2 Table). The estimated rate of bipolar disorder was 16.4% higher (95% CrI 5.8%–29.6%, *p_MCMC_* = 0.0044) in the most densely populated counties (Fig 3A). For major depression, a slight increase of 6% in the diagnosis rate (95% CrI 0%–12.4%, *p_MCMC_* = 0.05) was observed only among the worst air quality regions (Q7). We also observed a positive association with a small effect size between population density, urbanicity, and the rate of major depression diagnosis (see Fig 3D and S2 Table). Personality disorder was best predicted by land pollution (Alaska and Hawaii were not included in this analysis because we did not have matching high-resolution weather data). The regions with worst land quality (Q7) were associated with an estimated 19.2% increase (95% CrI 8.8%–29.9%, *p_MCMC_*<10^−4^) in the apparent rate of personality disorder (Fig 3C and S2 Table).

The apparent protective effect of pleasant weather days was high across all our target disorders and was highest for bipolar disorder in our analysis. The counties with the highest number of pleasant weather days (Q7) were associated with an estimated 21.8% decrease (95% CrI 16.8%–26.8%, *p_MCMC_*<10^−4^) in the rate of bipolar disorder (Fig 3A–3F). At first glance, it seems counterintuitive that across all studied psychiatric and neurological disorders, both mean numbers of pleasant and harsh days would appear to be associated with a protective effect in neuropsychiatric disorders (Fig 3A–3F). However, this is not a contradiction or error because, in the continental climate, the number of days with at least 4 pleasant hours is strongly correlated with the number of days with at least 4 harsh hours. In these places, the same day can contribute to both the pleasant and the harsh list (e.g., pleasant in the early morning or late evening and harsh at midday). Therefore, it is likely that one effect, possibly the protective days with harsh weather (keeping individuals indoors, away from environmental exposure to contaminated air and land), is causal, and another effect—the number of pleasant days—is driven by a secondary correlation.

Random effects at the state and county levels showed dissimilar distribution across all 6 disorders studied here. For example, random effects for Michigan, Missouri, New Mexico, and Georgia were consistently high, whereas those for South Dakota, Iowa, Wyoming, and North Carolina were consistently low (see Fig 3G and S4 Fig). There were relatively few counties in which the county-level random effect was consistently low or high. For example, several counties in Southern California were low: San Diego, Imperial, Orange, and San Bernardino. Likewise, several counties were consistently high: San Luis Obispo in California and Snohomish and King in Washington (see Fig 3H and S4 Fig).

### Sensitivity analysis for the association between air quality and bipolar disorder

In order to correct for multiple testing, we applied false discovery rate (FDR) correction to the *p*-values obtained from the regression analysis. The association between air quality and bipolar disorder remain statistically significant after FDR correction, whereas a previously observed weak association of major depression with only worst air quality regions (Q7) did not survive the multiple correction (S2 Table). We performed further sensitivity analysis to test the significant association observed between air quality and the rate of bipolar disorder in the US. A validation study of bipolar disorder’s diagnosis in hospital discharge registers suggests that the two-separate discharge diagnosis measure was sufficiently sensitive and specific for us to use in our epidemiological study [42]. We further validated our model by considering a subset of the population with at least 2 or more insurance claims diagnosed as bipolar disorder during the study period of 2003–2013. A total of 906,175 individuals (345,318 males and 560,857 females) met this criterion. Validating the model with this new criterion showed similar trends as reported above (S5A Fig and S6A Fig). Notably, air quality turned out to be the strongest environmental predictor of bipolar disorder. The regions with worst air quality (Q7) showed a 29% increase (95% CrI 16.4%–43.4%, *p_MCMC_*<10^−4^) in the apparent rate of bipolar disorder (see S5A Fig and S6A Fig). Lithium is often considered as a gold standard for treating bipolar disorder [43, 44]. We ran an additional model by redefining the bipolar disorder cohort to include individuals with a history of at least 1 dispensed prescription of lithium (37,964 individuals) in addition to those who had at least 1 insurance claim of bipolar disorder. The results and the trends from these models were comparable to the results reported earlier (S5B and S5C Fig). Random effects at the state and county levels showed dissimilar distribution across all neuropsychiatric disorders (see S6B and S6C Fig).

### Model validation and adjustment for spatial autocorrelation

The six neuropsychiatric disorders considered in this study showed variable degrees of spatial autocorrelation at the county level. These spatial dependencies could potentially artificially reduce variance in observations and inflate the effect size of the covariates, leading to biased parameter estimates. To probe the importance of the spatial dependency of outcomes, we tested both nonspatial and spatially explicit (conditional autoregressive [CAR]) models. Bayesian analysis of very large datasets with hierarchical mixed-effects models and spatial correction was computationally very expensive. Therefore, for this comparative analysis, we did not stratify data by age and gender groups, and therefore the models do not represent age- and sex-corrected estimates. Parameter estimates, analyses of residual spatial autocorrelation, and Bayesian posterior predictive checks were used to compare model performances. For the nonspatial model, we used a mixed-effect Poisson regression with the same exposure and covariates as used previously (except for age and sex) and measured random effects at the state and county levels. For the spatial model, we used county adjacency information (from the US Census Bureau) to design binary, first-order adjacency weight matrix and corrected for spatial autocorrelation using a CAR model. We tested for spatial autocorrelation among the residuals using Moran’s I test and found no autocorrelation among the residuals.

Comparing 2 versions of spatial analysis, we observed slight variations in some of the model estimates after accounting for spatial autocorrelation (S7 Fig and S3 Table). For bipolar disorder, the comparison of best (Q1) and worst (Q7) air quality regions suggests that risk increases by 29.7% (95% CrI 17.3%–43.3%) under nonspatial setting and by 23.4% (95% CrI 12.7%–36.3%) under spatial correction (S7 Fig and S3 Table). It should be noted that correction for spatial dependencies slightly reduced the estimated effect of air quality on the rate of bipolar disorder, but the association remains strong and statistically significant. On the other hand, a marginally higher rate of major depression (only among the worst air quality regions [Q7]) remained consistent across the models. After correcting for spatial autocorrelation, the estimated rate of personality disorder in the worst land quality regions (Q7) increased from 19.7% (95% CrI 9.4%–29.7%) to 25.9% (95% CrI 13.9%–37.7%) compared to the best land quality regions (Q1) (S7 Fig and S3 Table). In general, for all disorders, the correction of spatial dependencies slightly reduced the estimates for ethnicity, population density, and weather variables (S7 Fig and S3 Table). With leave-one-out cross-validation, the comparison of nonspatial and spatially explicit models suggests that the predictive performance decreases marginally in all 6 models after adjusting for spatial autocorrelation. We tested for spatial autocorrelation among the residuals by computing Moran’s I statistics and found no signs of spatial correlation in any of the models, suggesting that first-order binary adjacency weights were sufficiently able to eliminate spatial dependencies.

To further evaluate the robustness of the models, we split the data into 2 subsets (subset 1 and subset 2). For each state, we randomly assigned equal numbers of counties to both subsets. The 2 subsets included representative samples from 49 states (excluding Alaska and Hawaii), with subset 1 consisting of 1,532 and subset 2 consisting of 1,557 counties. For each neuropsychiatric disorder, we produced separate models from subset 1 and subset 2 and tested them against each other. In general, with few exceptions, the model estimates from subset 1 and subset 2 were mostly consistent and comparable (S8 Fig). The association between air quality and bipolar disorder remained significant in both the models. Importantly, model 1 suggested a 33.6% increase (95% CrI 16.1%–53.5%) and model 2 suggested a 29.6% increase (95% CrI 11.6%–50.7%) in the rate of bipolar disorder when comparing the worst air quality regions (Q7) with the best air quality regions (Q1). When tested against one another, the 2 independent models showed robust prediction capability, with Bayes R-Square for the bipolar disorder models as follows: subset 1 (0.989) when tested on subset 2 (0.95), and subset 2 (0.987) when tested on subset 1 (0.948). Models for other phenotypes similarly showed strong prediction strength when tested with independent datasets (S9 Fig). These independent model validations indicate robustness of the associations reported earlier in this study.

### Childhood individual-level exposure to air pollution and the risk of psychiatric disorders in Denmark

We used Danish national registers comprising all individuals born in Denmark between January 1, 1979, and December 31, 2002, who were alive and residing in Denmark at their 10th birthday (1,436,702 unique individuals) to study 4 psychiatric disorders: bipolar disorder, schizophrenia, personality disorder, and depression. We estimated air pollution exposure for all individuals from birth until age 10 and studied the association between childhood exposure to air pollution and 4 psychiatric disorders. We performed principal components analysis (PCA) on 14 air quality indicators to obtain a summarized measure of exposure to the air pollution (see S10 Fig, S11 Fig, and Methods for details). We transformed air pollution exposure into septiles, with Q1 representing the least exposure and Q7 representing the highest exposure to the air pollutants. It is important to highlight here that, though the general concept and pipeline are similar, the exposure composition and the statistical model used for the Denmark data analysis are technically different from the one used for the US analysis (see Methods for details). The high resolution of the Danish national registers made it possible to estimate the exposure to air pollution at the individual level—in contrast with the US data analysis reported earlier, in which the exposure is measured at the county level. These differences were primarily dictated by the availability and resolution of the data. Caution should be made in direct comparison of the results from cross-country analysis.

**Fig 4:**
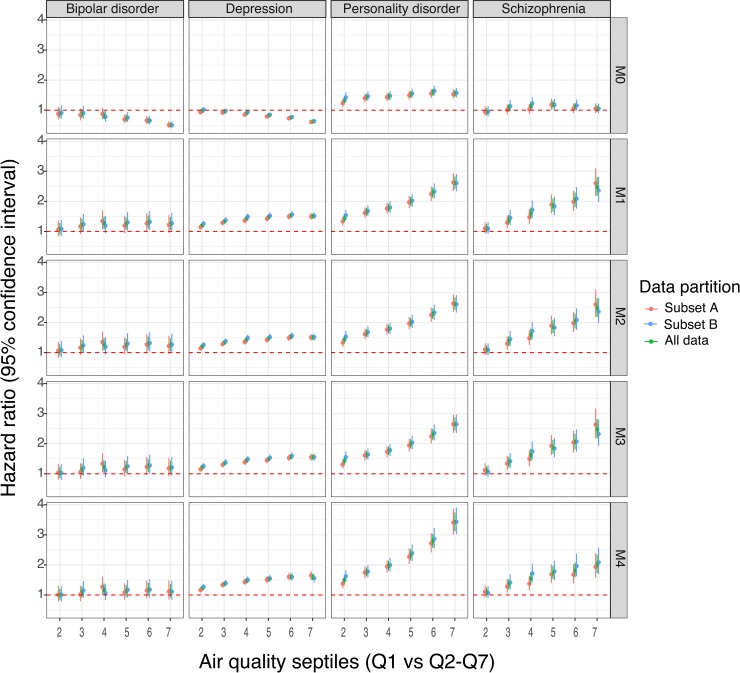
Association between air quality and the risk of psychiatric disorders in Denmark. The results from the Danish data analysis in which the individual-level estimates of air quality exposure are divided into septiles, with each septile representing approximately 200,000 individuals. Septile 1 (representing the least exposure) is used as a referent to compare disorder rates in the higher septiles for bipolar disorder, schizophrenia, personality disorder, and major depression. Higher septiles represent individuals with systematically higher exposure to low-quality air. Five different models (labelled M0–M4) were run for each phenotype, briefly as follows: M0: crude model with 7 air-quality–exposure groups; M1: M0 + calendar time using splines; M2: M1 + sex; M3: M2 but restricted to subset of population with no missing covariates; and M4: M3 + socioeconomic status + urbanization. Further, to cross-validate the models, whole data were split into 2 equal subsets (subset A and subset B), separate models were run on each subset, and the parameter estimates were compared. The figure shows estimates from subset A, subset B, and from the model using all the data. The underlying data for this figure can be found in S4 Table.

Results from the Cox regression models suggest that, for all 4 psychiatric disorders, the rate of disorders increases with increasing levels of exposure to air pollution. The estimated rate of schizophrenia was 148% higher (95% confidence interval [CI] 119%–180%, *p* < 2 × 10^−16^) among individuals in the group with the highest exposure to air pollution (Q7) compared with those with the least exposure (Q1, the referent group; shown in Fig 4 and S4 Table). The estimated rate of bipolar disorder was 29.4% higher (95% CI 9.4%–52.9%, *p* < 3 × 10^−3^) and 24.3% higher (95% CI 4.5%–47.9%, *p* < 0.014) in the exposure categories Q6 and Q7, respectively, compared with Q1. The strongest association was between air pollution and personality disorder, showing a 162% increase (95% CI 142%–183%, *p* < 2 × 10^−16^) in the disorder rate among category Q7 compared with category Q1. The estimated rate of major depression increased by 50.5% (95% CI 42.8%, 58.7%], *p* < 2 × 10^−16^) among the group with the highest exposure to air pollution (Fig 4). (Note: The complete within-group comparison of estimated rates can be found in Fig 4 and S4 Table.) The association between air quality and the risk of all 4 psychiatric disorders remained statistically significant even after correcting for multiple comparisons (see S4 Table).

To test the robustness of these model estimates, a cross-validation analysis was performed on the Danish dataset. The whole cohort was randomly partitioned into 2 equal-size subsets that were analyzed separately, and results of the analyses were compared (Fig 4). The two subsets provided nearly identical results.

### Harmonization of the US and Denmark data analysis

In the Denmark analysis, it did not make sense to aggregate data geographically by administrative region when individual-level data at a resolution of 1 square kilometer were available. We did run the analysis over the Denmark cohort using a Poisson model instead of Cox. The results were very similar to the initial Cox regression analysis, as shown in the Supporting Information (S12 Fig). To harmonize the analysis of data from 2 different countries, we adjusted the models built on the Denmark data for potential socioeconomic confounders such as urbanicity, parental educational levels, income, and employment status (all measured at an individual level on their 10th birthday). The information on these covariates was not readily available for the entire study population, so a subset of the dataset was used for the subsequent analysis. The results from the adjusted models were consistent and comparable to the results reported in the earlier models (see Fig 4 and S4 Table). Notably, by adjusting for socioeconomic confounders, the previously estimated rate of bipolar disorder slightly diminished and that of personality disorder increased, but the overall trend of association remained comparable.

The air quality index used in the US analysis (designed by the EPA) is a summary measure, obtained from the PCA of mean exposure to the 87 air quality indicators, whereas for Denmark, the exposure is a summary indicator of 14 air quality indicators modeled from birth until a patient’s 10th birthday. In an attempt to harmonize the 2 analyses, we performed a sensitivity analysis by using the same air quality indicator variables across the 2 studies. First, we recomputed the US county-level air quality index with a subset of 6 air components (carbon monoxide [CO], nitrogen dioxide [NO_2_], ozone [O_3_], particulate matter smaller than 10 μm [PM_10_], particulate matter smaller than 2.5 μm [PM_2.5_], and sulfur dioxide [SO_2_]) that were available for both the US and Denmark. With a mixed-effect Poisson regression model, we again observed a significant association between the air quality and risk of bipolar disorder in the US. The counties with the worst air quality (Q7) showed an estimated 11.6% increase in the rate of bipolar disorder (S13 Fig). Secondly, we reanalyzed Denmark data with the exposure estimated from 6 air components discussed above. The estimates from these models were again very similar and comparable. Specifically, the rate increase in the highest exposure group (Q7) compared to the least-exposure group (Q1) was as follows: bipolar disorder 31.4% (95% CI 7.4%–60.8%, *p* = 0.007), schizophrenia 104.3% (95% CI 76.3%–136.8%, *p* < 2 × 10^−16^), personality disorder 209.6% (95% CI 183.5%–238%, *p* < 2 × 10^−16^), and major depression 68.3% (95% CI 57.9%–79.4%, *p* < 2 × 10^−16^) (S4 Table). (Note that we present complete results of this analysis, for all 7 groups of environmental quality, in S4 Table.)

### Analysis of individual air quality indicators

In this observational study, we hypothesized that different pollutants interact with each other in a synergistic way that can be captured by PCA and represented by the first principal component (PC1) of variation over the 87 air quality indicators. A downside of this approach is that interpretation of the PCA in terms of air pollution is not necessarily straightforward. The full study of space of exhaustive combinatorial interactions among 87 environmental factors will be computationally intractable, especially in a setting of Bayesian multilevel mixed-effect regression. In addition, most of the air pollutants were multicollinear, preventing easy disentanglement of their individual contributions. We performed a mixed-effect Poisson regression analysis of the full collection of 87 US air quality indicators and identified several strong predictors of bipolar disorder prevalence: cyanide compounds (variable A_CN), acrolein (A_ Acrolein), acrylonitrile (A_C3H3N), bromoform (A_Bromoform), epichlorohydrin (A_ECH), polychlorinated biphenyls (A_PCBs), and vinyl acetate (A_ VyAc). The strongest predictors in a similar analysis conducted for individual air components in the Denmark dataset (across all 4 neuropsychiatric conditions) included nitrate (variable NO3_mean), ammonium (NH4_mean), sulfate (SO4_mean), EC (EC_mean), and organic carbon (OC_mean). Full results are available in S5 Table and S14 Fig.

## Discussion

The datasets representing US and Denmark populations in this study have different strengths. The US dataset is 2 orders of magnitude larger than the Danish dataset but is at a county level, whereas the Danish dataset allows for the computation of individual-level pollutant exposure during the first years of a patient’s life with a spatial resolution of 1 square kilometer. In the US data, patient early-life trajectories are not known, and we had to estimate exposure using county-level pollution measurements, assigning patients to their county of residence during the period recorded in the insurance data. As some US counties are very large, we should expect that the estimated quality for individual exposure would be degraded for such counties. US environmental data have additional variables (e.g., land, built, and water quality) that are not available for this study for Denmark. Data from Denmark included all eligible individuals, with information from all psychiatric treatment facilities within the country in the context of universal healthcare. The potential risk for selection or information bias is thus reduced.

Just as the 2 very large national datasets used in this study have different strengths, they also have divergent limitations and biases. They reflect life in different cultures, with diverging approaches to healthcare, population tracking, and environmental monitoring. For example, apparent disease prevalence is affected by the ascertainment biases, diagnostic biases, social stigma, and healthcare practices specific to a geographic area, and the variability across the racial/ethnic categories in the data should be read with these qualifications in mind. Given these many differences, it is all the more significant that the patterns we see in findings across 2 diverse countries are consistent for bipolar disorder. The Denmark analysis suggests that poor air quality during the initial years of an individual’s life increases the risk of all 4 psychiatric disorders studied here (bipolar disorder, schizophrenia, personality disorder, and major depression). In the US data, we see a similar trend for bipolar disorder (and to some extent for major depression) as that in Denmark, but the signal for schizophrenia and personality disorder is absent. It is likely that this difference is due to the limited resolution of the pollutant exposure estimates for the US data. It is also possible that this difference is partially caused by differences in study design, exposure composition, or country-specific genetic variation. Our US analysis was, by necessity, focused on association of disease with recent influence of pollution, while Denmark data allowed for evaluating corresponding association with cumulative long-term effect.

In our exploratory analysis, we found that poor air quality is associated with apparently higher rates of bipolar disorder and major depression in both US and Danish populations. Air pollution is a complex and variable mixture of small particulate matter (PM), gases, metals, and organic contaminants generated by transport vehicles, industrial activity, and fires. To quantify air pollution in the US, we used the EPA air quality index, which is a summary measure, obtained from the PCA of 87 potential air pollutants. These pollutants include PM_10_ and PM_2.5_, as well as diesel emissions and NO_2_, itself often used as a proxy measure of air pollution, and organic substances such as polycyclic aromatic hydrocarbons (PAHs). There are multiple substances that contribute to the PC1 of the air pollution index shown in S15 Fig. Because multiple pollutants are collinear in their presence, we are unable to narrow the list of “suspect” causal pollutants to a specific compound. It is likely that multiple pollutants contribute to deleterious effects on the human nervous system in an additive or synergistic way. It is also possible that measured pollutants serve as surrogate variables to an unmeasured pollutant that causally affects human disorders. Ultrafine PM (<100 nm) and nanoscale PM (<200 nm), for example, are not separately assessed in the EPA air quality index yet are likely to track other indicators. Noise pollution likewise is outside the scope of these indices but is likely to track other indicators of vehicular and industrial emissions [45].

Growing evidence from human, animal, and in vitro studies demonstrates that airborne pollutants target the brain and are implicated in neurological and psychiatric disorders etiology [46–49]. Yet bipolar disorder’s and depression’s links to air pollution have not been examined to the same degree as other environmental factors such as psychosocial stressors; at the same time, studies of air pollution and the central nervous system have focused on disorders of neurodevelopment and aging such as autism and Alzheimer disease. The patterns uncovered in our data underline the potential importance of the physical milieu to bipolar disorder and depression research.

### Potential mechanisms

The associations detected in this observational study necessitate an explanation via likely biological mechanisms linking environmental exposures to neurological and psychiatric disorders. The most causally convincing studies involve experiments with animals. Significantly, a growing number of experimental animal studies tie environmental factors to inflammatory and cytotoxic damage to neural tissues and to psychiatric disorders. Below, we highlight those studies lending mechanistic insight into potential causal pathways underlying our observed associations.

In an early seminal study, healthy feral dogs chronically exposed to traffic-related pollution were studied by detailed analysis of tissue pathology [46]. Exposed dogs showed marked increases in cytopathological, immunological, and genetic damage responses in the lung and nasal epithelium, blood-brain barrier, and cortical and subcortical cells. Three pathways by which PM is likely to affect the brain were suggested, as follows. (A) Indirect transport of pollutants via the lungs leads to systemic inflammation. Fine PM first induces respiratory tract inflammation, which then leads to systemic inflammation of peripheral sensory nerves. This results in the production of brain cytokines, activation of microglia, and genomic oxidative damage; (B) direct transport by way of intravascular brain macrophages, the downstream effects of which are the same as the first pathway; and (C) direct transport of pollutants to the brain via nasal respiratory damage. In this pathway, olfactory neurons transport fine PM directly to the brain, producing direct toxic damage to the limbic system and brain degeneration due to oxidative stress.

All 3 pathways of brain exposure to pollution are likely to be present in humans, supported by postmortem brain tissue studies indicating the physical presence of vanadium and nickel PM from air pollution, as well as evidence of microglial activation and neuroinflammation [50]. Recent rodent model studies similarly point to both systemic and nose-to-brain routes for pollution impacts on the brain. Analysis of rats exposed to different fractions of airborne PM captured from Riverside, California, showed that 1- to 3-month exposures to PM_2.5–10_ resulted in both metal accumulation in the brain and up-regulation of genes in inflammatory cytokine pathways as well as some linked to tumorigenesis [47]. In another study, healthy 4-week-old male mice were exposed to urban-like ambient fine airborne PM pollution in laboratory conditions for 10 months [48]. The animals were subjected to behavioral tests and a battery of analyses. Analysis of tissues of the exposed animals revealed inflammation of brain tissues [51], especially in the hippocampus. Moreover, exposed animals showed signs of cognitive impairment in spatial learning and memory and depression-like behavioral symptoms.

### Neuroinflammation, excitotoxicity, and psychiatric disorders

Thus, is it clear that both systemic and direct nose-to-brain routes generate neuroinflammation and oxidative stress. This is important for the current analysis because neuroinflammatory and excitotoxic processes have been linked to psychiatric disorders generally and bipolar disorder in particular in animal models and human patients. Because microglia shift from a quiescent to an activated state and secrete pro-inflammatory cytokines and reactive oxidants when the brain is injured or their microenvironment is perturbed, the activation of microglia and the cytokines they produce have come under particular scrutiny. Analysis of postmortem frontal cortex tissue from bipolar disorder patients compared to matched controls showed higher mRNA and protein levels of biomarkers of neuroinflammation, as well as signs of increased excitotoxicity [52]. Studies of neuroinflammatory markers in cerebrospinal fluid show increased levels of interleukin-8 (IL-8) in euthymic bipolar patients versus controls, which also correlated with lithium treatment [53]. A study of heightened neuroinflammation markers in relation to clinical outcomes was inconclusive; the authors hypothesize that these markers may indicate a vulnerability to the disorder rather than a reflection of disease course [54].

Depression has also been linked to neuroinflammation and microglia dysregulation, for example, after traumatic brain injury [55]. Animal models of depression generated using chronic stress frameworks such as repeated social defeat or foot-shock show activation and increased branching of microglia and related inflammatory markers, supporting the hypothesis that microglial homeostasis perturbations are part of depression’s underlying disease process [56]. Interestingly, stress-induced depression may also be associated with microglial decline and senescence [57].

As noted above, there is a strong tendency to equate “environment” in neurological and psychiatric disorders with psychosocial family milieu or infectious disease. Our results indicate that the physical environment, in particular air quality, warrants further attention in research seeking to elucidate environmental contributors to neurological and psychiatric disease risk. In conclusion, we observed a strong positive association between exposure to environmental pollution and an increase of prevalence in psychiatric disorders in affected patients. Converging data points to neuroinflammatory mechanisms linking environmental compounds to their putative psychiatric consequences. However, these strong associations do not necessarily mean causation; further research will be needed to assess whether air pollution’s neuroinflammatory impacts share common pathways with other stress-induced conditions.

## Methods

### Ethics statement

The University of Chicago IRB determined that the study is IRB exempt, given that patient data in both countries were preexisting and de-identified.

We first present the materials and methods used for the analysis of the US cohort followed by a similar description for the Danish cohort.

### Data sources for the US analysis

We used the IBM MarketScan health insurance claims database [34] that includes both inpatient and outpatient claims, medical procedures, and prescription medications for 151,104,811 unique patients for the period of 2003 to 2013. The IBM MarketScan health claims database is a compilation of patient records from over 100 insurance carriers and large, self-insuring companies in the US. The approved claims are linked across years and geocoded at the county level. In addition to diagnostic and prescription medicine claims, records include patient’s age, sex, and geolocation aggregated to the county level. Individual-level race/ethnicity was not available in the MarketScan database; therefore, 2010 US Census [58] data were used to link county-level percent racial distributions for the following groups: American Indian, Asian, black Hispanic, black non-Hispanic, Pacific Islander, white Hispanic, and white non-Hispanic.

We used the EQI [59, 60], a summary measure constructed by the US EPA, to represent the environmental quality of all counties in the US. The EQI represents 5 US county-level environmental domains (air, water, land, built, and sociodemographic) that are further incorporated into a single index for the years 2000 to 2005. Data sources and the construction of the EQI have been described in detail by the EPA [59, 60]. Briefly, 187 data sources were evaluated for inclusion; those that were retained for their data quality and availability at the county level for the entire US enabled the use of 219 unique variables across each of the 5 domains: air (87), water (80), land (26), built (14), and sociodemographic (12). A PCA, performed individually on each domain, produced 5 domain-specific indices for the corresponding environmental domain. For most states, the water quality showed very little variation within the state. Two additional datasets, DAYMET [61] and North American Land Data Assimilation Systems [62], were used to link weather-related variables.

### Disease outcomes

The IBM MarketScan dataset includes time-stamped patient treatment episodes with individual patient diagnoses. These treatment episodes, both inpatient and outpatient, are represented with ICD-9 codes; patient sex and age were also recorded, with each patient visible from 1 to 11 years (during 2003 to 2013) in the dataset. The de-identified version of the dataset used here also contains limited data about the geographic location of patients at the US county level, which helped us in estimating the environmental exposures.

We studied 4 psychiatric and 2 neurological disorders: bipolar disorder, major depression, personality disorder, schizophrenia, epilepsy, and Parkinson disease, each disorder defined by sets of specific ICD-9 codes. We framed our analysis around bipolar disorder and performed comparative analysis with schizophrenia, Parkinson disease, personality disorder, major depression, and epilepsy. The outcome, bipolar disorder, observed in either inpatient or outpatient settings, was defined as patients with a bipolar disorder claim over the period of 2003–2013, identified in MarketScan database by ICD-9 code 296.x (where x refers to 296.00, 296.01, 296.02, 296.03, 296.04, 296.05, 296.06, 296.10, 296.11, 296.12, 296.13, 296.14, 296.15, 296.16, 296.40, 296.41, 296.42, 296.43, 296.44, 296.45, 296.46, 296.50, 296.51, 296.52, 296.53, 296.54, 296.55, 296.56, 296.60, 296.61, 296.62, 296.63, 296.64, 296.65, 296.66, 296.7, 296.80, 296.81, 296.82, 296.89, 296.90, and 296.99). Similarly, we used ICD-9 codes to capture a broad definition of schizophrenia (295.x, 298.x, and 293.82 for psychotic disorder with delusions and 293.82 for psychotic disorder with hallucinations); 332.x for Parkinson disease; 301.x for personality disorder; 296.2x, 296.3x, and 311 for major depression; and 345.x for epilepsy. When we refer to these 6 conditions, we are explicitly referring to data captured by IBM MarketScan, which is the treated prevalence inferred from US insurance claims; because the data was potentially influenced by reporting biases, we refer to the IBM MarketScan disease rates as raw rates, to be further adjusted for confounders.

### Environmental risk factors

The environment appears in our model as 3 sets of variables at the US county level: (i) quality of air, water, land, and “built” environment (e.g., amount of vehicular traffic, transit access, and pedestrian safety). These data were divided into septiles, with septile 1 representing the best and septile 7 the worst environmental quality (Fig 1A–1D); (ii) weather indices split into a number of days with at least 4 hours of pleasant weather (defined according to the design standards for climate-control buildings), and number of days with at least 4 hours of harsh (either too hot or too cold) weather (Fig 1E–1F). This last group of factors is useful in dissecting the outdoor environment’s positive (open-air activities) and negative (pollution) influences; and (iii) Population density and urbanicity status (S1 Fig), known risk factors for many psychiatric disorders.

The domain-specific EQIs for air, water, land, and built quality were used as the main exposure variables. We conducted analyses using septiles of the domain-specific EQIs to compare higher septiles (7 = worse quality) to the lowest septile (1 = best quality) across all US counties. Our model also included county-level median income, population density, and the percentages of poor, insured, and urban population (see S1 Fig). In this study, we made use of 2 county-level weather variables. The first weather variable was a measure for “good days” that indicated whether at least 4 hours in a diurnal cycle were in a “comfort zone,” defined as a 4-point patch with vertices in temperature and humidity space (temperature [18 °C, 27 °C, 27 °C, 18 °C] and specific humidity [6.71%, 8.85%, 13.85%, 10%]). The second weather variable was a measure of “bad days” that indicated whether at least 4 hours in a diurnal cycle were in an “extremely uncomfortable zone,” defined as < −5 °C or > 35 °C. Good days and bad days are not mutually exclusive; for each, the number per year was averaged over the years during the period 2003–2012. The 4-hours-per-day measure was selected as a reasonable time interval that can be used for outdoor activities. We did not perform a maximization of association of weather predictors and disorder rates to select these indices.

### Statistical analysis

We used a mixed-effects regression, modeling counts of neurological and psychiatric disorders per (age and sex) group per county with a Poisson distribution, where the logarithm of the Poisson rate is defined by a linear combination of predictors and random effects. We implemented this approach using Markov chain Monte Carlo (MCMC) algorithms. Time in our regression analysis was handled in the following way: for each county/demographic group, we computed the “offset,” which we defined as the total number of patients in a given county, visible to the dataset within the 11-year interval (2003–2013). The IBM MarketScan database comprised a total of 151,104,811 unique individuals; 79.48% of them had at least 1 disease-specific claim recorded in the dataset, and 53.89% had at least 1 medication-specific claim. Overall, about 17.61% of the individuals did not have any disease- or medication-related claims and were considered as healthy insured individuals. Our analysis consisted of a subset of the IBM MarketScan database restricting to those individuals whose place of residence was known at the US county level (Table 1). We used a county-identifiable IBM MarketScan population of 100,316,345 for computing the offset. For each disorder and county, we computed the unique number of patients with a disorder diagnosis; we again stratified patients by sex and age within the county. We then used both offsets and counts of patients with the disorder diagnosis in the mixed-effect regression model. Fixed-effect covariates included individual-level sex (female as a referent); individual-level age category (0–5 [referent], 6–17, 18–30, 31–40, 41–50, 51–65, and 66 and above); county-level race/ethnicity; median income; the quality of air, water, land, built, and weather; and percentages of poor and insured population.

We transformed county-level sociodemographic variables, population density, weather variables, and racial composition into septiles such that higher septiles represent higher numbers and percentages. Similarly, we transformed air, water, land, and built EQIs into septiles such that higher septiles represent worse quality. We modeled these variables comparing different septiles to estimate relative prevalence ratios (PRs) and CrIs. In addition to the fixed effect, random effects were measured at the county and the state levels. State-level random effects likely absorbed state-specific differences in both disorder reporting and true prevalence, whereas the county-level random effects can be thought of as residual variations not explained by our fixed-effect predictors and state-level random effects. We ran several models and compared them by using deviance information criteria. For each model, 120,000 MCMC iterations were run with a burin of 20,000 and a thinning interval of 10. The statistical analyses were conducted in R version 3.4.0 and RStudio version 1.0.143 using a mixed-effect regression model implemented in MCMCglmm package [63].

### Cross-validation and adjusting for spatial autocorelation

The 6 neuropsychiatric disorders considered in this study showed variable degrees of spatial autocorrelation between the observations. These spatial dependences have tendency to artificially reduce variance in observations and inflate the effect size of covariates, leading to the biased estimates from regression analysis. To uncover the consequences of overlooking this spatial dependency component, we tested both nonspatial and spatially explicit (CAR) models. The mixed-effects hierarchical models with big data and Bayesian framework were computationally very expensive with no guarantee of convergence, so we reduced the model complexity and computation time by aggregating (previously age- and sex-stratified) disease data at the US county level; for each county, we obtained the count of individuals with the respective disease diagnosis and the total number of individuals at risk. In other words, for comparing spatially explicit and nonspatial models, we did not stratify data by the age and gender groups, and hence the models do not represent age- and sex-corrected estimates. Parameter estimates, analyses of residual spatial autocorrelation, and Bayesian posterior predictive checks were used to compare model performance.

We used county adjacency information (from the US Census Bureau) to a design binary, first-order adjacency weight matrix that has been previously reported to be a good choice for Bayesian models [64]. Neighbors are defined as counties that share a common boundary, and a weight of 1 was assigned if the 2 counties are neighbors and 0 otherwise. We used mixed-effects Poisson regression with the same exposure and covariates as used previously (except for age and sex) and measured random effects at the state and the county levels. In addition, we corrected for spatial autocorrelation using a CAR model. Because we used a slightly different implementation of the model in contrast to the one mentioned previously, we provide comparative analysis of nonspatial and spatially explicit models to make a fair comparison. We evaluated the spatial autocorrelation among the residuals using Moran’s I test.

Thereafter, we validated nonspatial and spatially explicit models using leave-one-out cross-validation. To further evaluate the robustness of the models, we split the data into 2 subsets (subset 1 and subset 2), produced independent models from each subset, and tested them against each other. In order to have a complete state-level sample representation, we randomly picked a similar number of counties from each state and assigned them to either of the subsets. Subset 1 and subset 2 represented 1,532 and 1,557 counties, respectively (note that we did not match counties in subset 1 and 2 based on any predictor variable or covariates such as urbanicity, environmental quality, weather quality, etc.). We evaluated the model predictive performance by using Bayes R-Square [65].

For each model, we ran 4 MCMC chains each with 3,500 iterations and burin of 1,500. We used R version 3.4.0 and RStudio version 1.0.143 using a mixed-effect regression model with spatial autocorrelation structure implemented in BRMS package [66].

### Key assumptions

This is an observational study that has several limitations and assumptions. The results reported in this study should be interpreted with these assumptions in mind. First, the “environment” in our analyses refers to the outdoor environment. The EPA has converted these outside environment measurements to EQIs, where the use of EQI as a measure of exposure assumes that exposure to “environment” is consistent in all individuals but the extent of environmental exposure was not assessable. Second, we generated the data corresponding to the observed counts of people within each county diagnosed with bipolar disorder, schizophrenia, Parkinson disease, personality disorder, major depression, and epilepsy using a Poisson process with the rate varying over the counties. The logarithm of Poisson rate of disease rate depends on a linear combination of fixed and random effects. Third, we used the count data from the insurance claims resulting in diagnosis of specific conditions in which we did not take disease severity into account. Finally, the MarketScan database has claims ranging from the year 2003 to 2013, and the EPA’s EQI database was constructed based on the counties’ data from 2000 to 2005. Using the air quality index, we grouped counties into septiles, with each septile representing slightly over 400 counties. We assumed that the county-level environmental quality (in terms of septiles) did not change drastically between 2006 and 2013.

### Validation on Danish national cohort

#### Study population

Since 1968, the Danish Civil Registration System [41] has maintained information on all residents, including sex, date of birth, continuously updated information on vital status, and a unique personal identification number that can be used to link information from various national registries. The study included data about all Danes who were born in Denmark between January 1, 1979, and December 31, 2002, and were alive and residing in Denmark at their 10th birthday (*N* = 1,436,702) [41]. Individuals who were born between the two dates but emigrated or died before their 10th birthday were excluded from the analysis. In addition, there were 1,628 (0.1% of the total study population) individuals for whom there was no information regarding exposure to air pollution who were excluded from the analysis. The final study population consisted of 1,435,074 individuals who were followed from their date of birth until the end of 2016.

#### Exposure estimation

The integrated Danish air quality dispersion modelling system THOR [67, 68] was used to model the atmospheric concentration of 14 compounds; CO, elemental carbon (EC), organic carbon (OC), ammonium (NH_4_), NO_2_, nitrate (NO_3_), nitrogen oxide (NO_X_), O_3_, PM_10_, PM_2.5_, sea salt, secondary inorganic aerosols (SIAs), SO_2_, and sulfate (SO_4_) for the period of 1979 and onwards with a spatial resolution of 1 km × 1 km. The THOR system is a coupling between the regional model den Danske Eulerske Hemisfæriske Model (DEHM) [69, 70] covering the Northern Hemisphere and the Urban Background Model (UBM) covering Denmark with a spatial resolution of 1 km × 1 km. For this area, high-resolution emission data based on the SPatial high REsolution Distribution (SPREAD) model for emissions to air [71] were included. The model simulations cover the period from 1979 and onwards. We summed the daily mean concentration of 14 environmental compounds at each individual’s residential address (extracted from the 1 km × 1 km dataset) from birth until their 10th birthday and subsequently divided the result by the total number of daily data points available to get a mean individual-level exposure to the air pollution. The completeness of Danish data was very high; indeed, a total of 1,401,515 persons (97.7%) had information on exposure measurements available on 90% or more of the days from birth until their 10th birthday.

In accordance with the design of the US EPA’s air quality index [59], we performed a PCA on the mean individual-level exposure to the 14 environmental compounds for complete study population and retained the PC1 as being representative of the individual-level exposure to air pollution (S10 Fig and S11 Fig). We further divided PC1 into 7 groups and compared psychiatric disorder rate among individuals in each of the 6 highest PC1 groups (Q2–Q7), with the rate among individuals in referent group Q1 (Fig 4).

#### Key assumptions

All the atmospheric components, with exception of O_3_ and sea salt, had positive loadings on PC1 (S10C and S10D Fig). In other words, 12 of the 14 compounds had a positive relationship with PC1, whereas O_3_ and sea salt had a negative relationship with PC1. Though PC1 is a good representative for most of the putative pollutants, caution should be made in interpreting the results reported in this study keeping the inverse relationship of O_3_ and sea salt with PC1 in mind.

We observed a decrease in mean concentration levels of all measured air compunds in Denmark over time, except for O_3_ and sea salt, which showed an increase over time (see S11 Fig). This is in line with the overall decreasing trend in the anthropogenic emissions of the main pollutants in Europe (see, e.g., https://www.eea.europa.eu/data-and-maps/indicators/main-anthropogenic-air-pollutant-emissions/assessment-4). Though we measured exposure for all individuals from birth to age 10, individuals born earlier were more likely to have been exposed to higher levels of air pollutants because overall pollution levels were higher in Denmark during the earlier years. This trend is reflected in the PCA as well; elderly subjects were generally assigned to higher scores on the PC1 axis.

Note also that the low-quality air exposure for the US study was estimated from 87 different compounds, while the exposure for Denmark study included estimates from 14 compounds. There were 6 air components in common (CO, NO_2_, O_3_, PM_10_, PM_2.5_, and SO_2_). We performed a separate analysis on all air compounds available to us and on mutually common components between the 2 counties.

#### Disease outcomes

Information on 4 psychiatric disorders (bipolar disorder, schizophrenia, personality disorder, and major depression) was obtained from the Danish Psychiatric Central Research Register (PCRR) [72], which contains records of all the admissions to psychiatric inpatient facilities since 1969 and visits to the outpatient psychiatric departments and emergency departments since 1995. The diagnostic system was based on the Danish modification of the ICD-8 from 1969 to 1993 and ICD-10 from 1994 onwards. The following diagnostics codes were used to obtain the patient records: bipolar disorders (ICD-10 codes F30–F31; ICD-8 codes 296.19, 296.39, and 298.19), schizophrenia (F20; 295.x9 excluding 295.79), personality disorders (F60; 301.x9 [excluding 300.49], 305.x9, 305.68, and 307.99), and major depression (F32–F33; 296.09, 296.29, 298.09, and 300.49). The outcomes listed were patients having at least 1 diagnostic visit related to bipolar disorder, schizophrenia, personality disorder, or major depression. The total person-years of follow-up for the 4 psychiatric disorders in the Danish data analysis were as follows: 21,954,767 for bipolar disorder, 21,688,539 for depression, 21,761,562 for personality disorder, and 21,913,828 for schizophrenia.

#### Statistical analysis

A PC1 score representing the relative cumulative scores of an individual’s exposures to air pollution was divided into septiles, with each septile representing slightly over 200,000 individuals. We performed a statistical analysis by using Cox proportional hazards regression models with age as the underlying time scale (each person was compared only to persons of the exact same age). We employed the models, which were adjusted for sex and birth date using splines, to compare the disorder rate in referent group Q1 to the rest of the groups (Q2–Q7) separately for each of the 4 mental disorders. The analyses of Denmark data were performed on the secured platform of Statistics Denmark using the packages “survival” [73] and function “coxph” on R version 3.4.3 and RStudio version 1.1.383.

### Harmonization of US and Denmark data analysis

In order to make US and Denmark data analysis more comparable, we first ran additional models on the Denmark data by including potential socioeconomic confounders into the models, such as urbanicity, parental educational level (primary school, high school/vocational training, and higher education), parental labor market affiliation (employed, unemployed, and outside workforce for other reasons), and household income (all measured at an individual level on their 10th birthday). The information on all covariates was only available for a subset of the population (*n* = 1,336,204; 93.1% of the total). We therefore performed these additional analyses for all 4 psychiatric disorders only in this subset. We again divided the covariates into different groups and observed the relative change in disorder rate among these groups.

To further harmonize the analysis from the two different countries, we ran a version of analysis keeping only air pollutants that were measured in both the US and Denmark. The EPA air quality index used in the US analysis is a summary measure, obtained from the PCA of mean exposure to the 87 potential air pollutants, whereas for Denmark the exposure is a summary measure of 14 pollutants modelled from birth until a patient’s 10th birthday. A subset of 6 air pollutants (CO, NO_2_, O_3_, PM_10_, PM_2.5_, and SO_2_) were available in both the US and Denmark. For the US, we reconstructed the county-level air quality index using the measured levels of these 6 air pollutants, and for Denmark, we recomputed the individual-level exposure to the above 6 air pollutants, both through the PCA as discussed earlier. After harmonizing the exposure composition, a mixed-effect Poisson regression model was used for the US data analysis, and a Cox regression was used for the Danish data analysis in a similar manner as discussed previously. The results were compared by matching the psychiatric disorder rates in the referent group (Q1) to the groups with systematically higher exposure (Q2–Q7) to the air pollution.

Both datasets were, in principle, amenable to analysis with the same methodology, such as Cox or Poisson regression. The major practical difference between these models was that the Cox regression (or proportional hazards regression) represents subjects in the study separately, with 1 row per individual. This representation works well for smaller datasets, such as the whole Denmark population, but is practically intractable for very large samples, such as the US dataset of over 150 million unique individuals. Poisson regression is similar to Cox regression in interpreting the risk associated with individual factors but allows for pooling individuals with the same characteristics (age, sex, county, state of disease) into one group, thus compressing data and making it manageable for practical computation. For that reason, we applied Cox regression to the smaller Denmark dataset but used Poisson regression with the US data. For comparison of the 2 analyses, we also ran Poisson regression analysis over the Denmark dataset; the results were virtually identical to the Cox regression version.

### Data availability statement

IBM MarketScan databases are available to purchase by Federal, nonprofit, academic, pharmaceutical, and other researchers. Use of the data is contingent on completing a data use agreement and purchasing the data needed to support the study. More information about licensing the IBM MarketScan databases is available at https://www.ibm.com/us-en/marketplace/marketscan-research-databases.

Access to individual-level Denmark data is governed by Danish authorities. These include the Danish Data Protection Agency, the Danish Health Data Authority, the Ethical Committee, and Statistics Denmark. Each scientific project must be approved before initiation, and approval is granted to a specific Danish research institution. Researchers at Danish research institutions may obtain the relevant approval and data. International researchers may gain data access if governed by a Danish research institution having needed approval and data access.

## Supporting information

S1 Data Complete study data used for the US analysis. (XLSX)

S1 Fig US county-level sociodemographic covariates. (TIF)

S2 Fig US state-level disease prevalence. (TIF)

S3 Fig US county-level disease prevalence. (TIF)

S4 Fig County- and state-level random effects in the US. (TIF)

S5 Fig Regression results of bipolar disorder sensitivity analysis in the US. (TIF)

S6 Fig Random effects from bipolar disorder sensitivity analysis in the US. (TIF)

S7 Fig Adjustment for spatial autocorrelation in the US analysis. (TIF)

S8 Fig Cross-validation of US models by splitting data into 2 partitions. (TIF)

S9 Fig Predictive performance of US cross-validation models. (TIF)

S10 Fig Air quality and exposure estimation for the Danish national cohort. (TIF)

S11 Fig Trends in air quality over time in Denmark. (TIF)

S12 Fig Comparison of results from Cox and Poisson models in Denmark. (TIF)

S13 Fig Regression estimates with reduced exposure to 6 air pollutants in the US. (TIF)

S14 Fig Regression estimates for individual air pollutant exposure in Denmark. (TIF)

S15 Fig US EPA county-level air quality and the rate of bipolar disorder in the US. PCA of EPA air quality measurements to produce air quality index. EPA, Environmental Protection Agency; PCA, principal components analysis.
(TIF)

S1 Table Prevalence of neuropsychiatric disorders from various published resources. (DOCX)

S2 Table Regression results from the US data analysis. (XLSX)

S3 Table Regression results from US data with nonspatial and spatially explicit models. (XLSX)

S4 Table Regression results from Denmark data analysis. (XLSX)

S5 Table US and Denmark regression results with individual air pollutants. (XLSX)

## Abbreviations

ADHD: attention deficit-hyperactivity disorder
CAR: conditional autoregressive
CDC: Centers for Disease Control and Prevention
CI: confidence interval; CrI, credible interval
DEHM: den Danske Eulerske Hemisfæriske Model
EC: elemental carbon
EPA: Environmental Protection Agency
EQI: Environmental Quality Index
FDR: false discovery rate
ICD-9-CM: International Classification of Diseases, Ninth Revision, Clinical Modification
IL-8: Interleukin-8
MCMC: Markov chain Monte Carlo
NIH: National Institutes of Health
NIMH: National Institute of Mental Health
OC: organic carbon
PAH: polycyclic aromatic hydrocarbon
PC1: first principal component
PCA: principal components analysis
PCRR: Psychiatric Central Research Register
PM: particulate matter
PM10: particulate matter smaller than 10 µm
PR: prevalence ratio
SIA: secondary inorganic aerosol
SPREAD: SPatial high REsolution Distribution
UBM: Urban Background Model
