# Correction: Environmental pollution is associated with increased risk of psychiatric disorders in the US and Denmark

**DOI:** 10.1371/journal.pbio.3000513

**Published:** 2019-10-02

**Authors:** The PLOS Biology Staff

## Notice of republication

This article was republished on September 16, 2019, to add an Editor’s Note that was accidentally omitted during production. The publisher apologizes for this error.

Please download this article again to view the correct version.

The Editor’s Note for this article serves to contextualize publication by highlighting any conflict in the reviewers’ opinions, or caveats that should be considered. It also directs the reader to the associated Primer for the article which adds further context and a complementary expert perspective (https://doi.org/10.1371/journal.pbio.3000370). Please see the PLOS Biology Staff Editors’ Editorial for further information about this initiative: https://doi.org/10.1371/journal.pbio.3000234
