# Supplementary figures and images for: Environmental pollution is associated with increased risk of psychiatric disorders in the US and Denmark
Source: PLoS Biol. 2019 Aug 20;17(8):e3000353. doi: 10.1371/journal.pbio.3000353 (PMC6701746; doi:10.1371/journal.pbio.3000353)

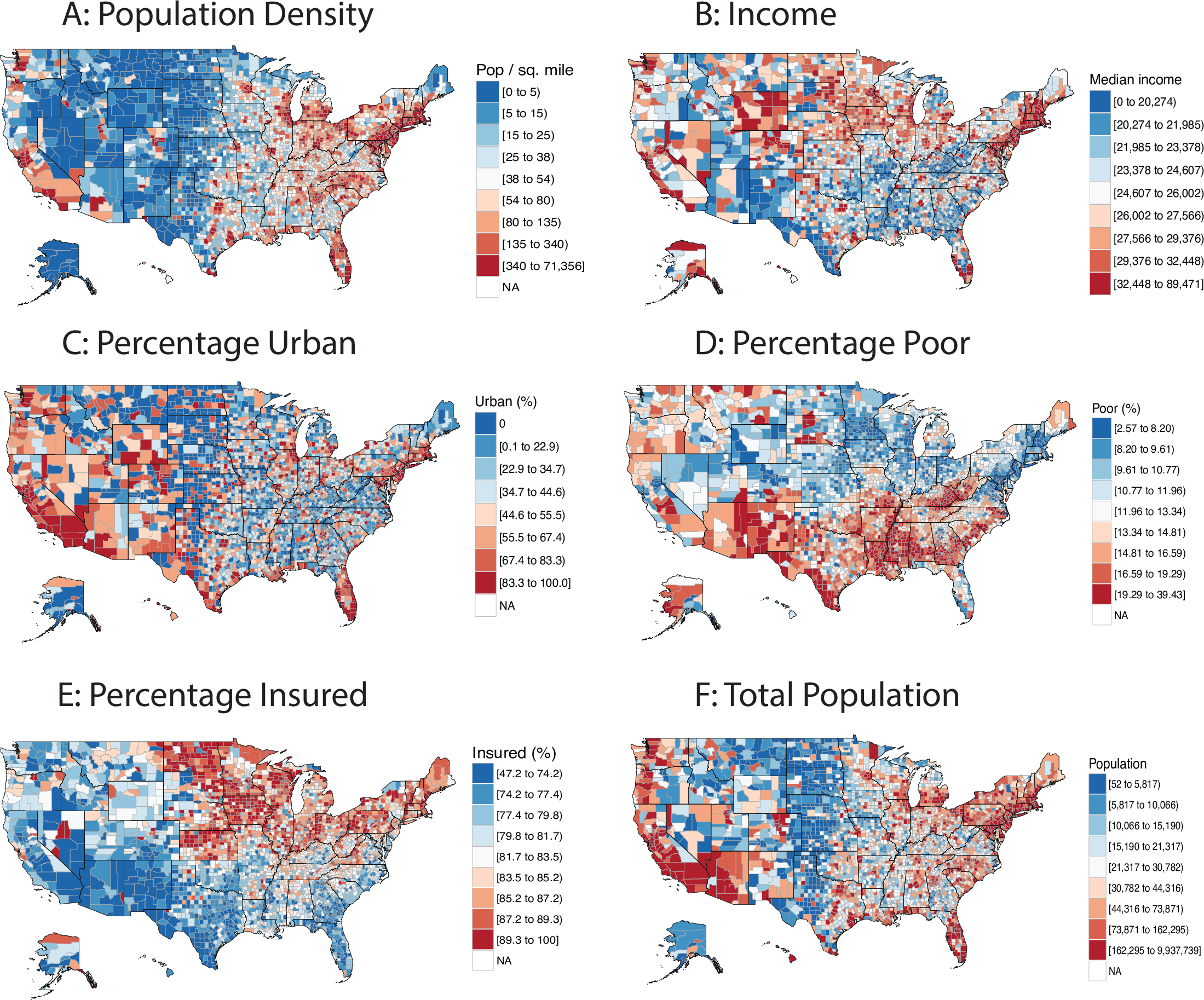

Supplement: S1 Fig — (TIF) [file pbio.3000353.s002.tif]

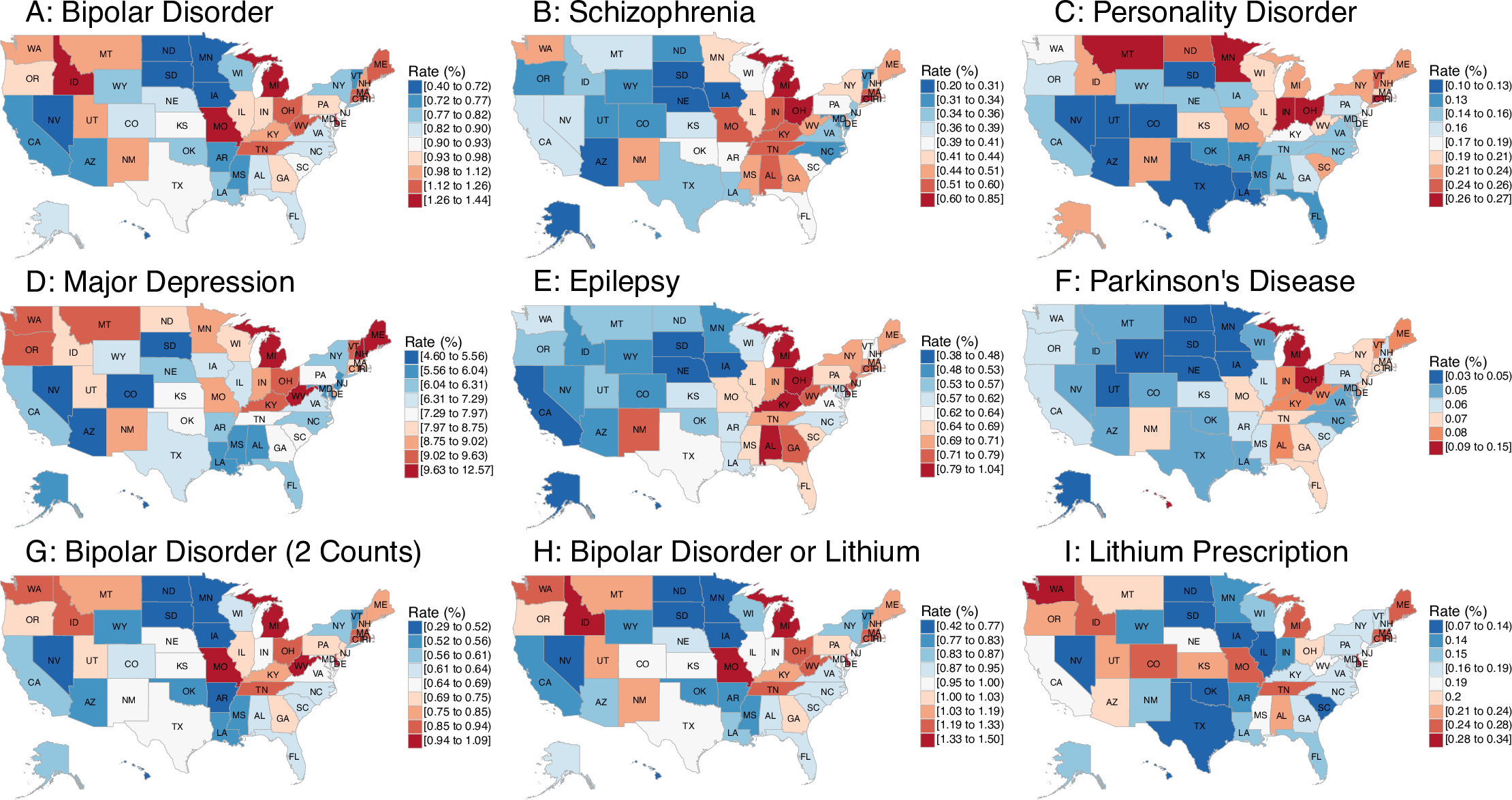

Supplement: S2 Fig — (TIF) [file pbio.3000353.s003.tif]

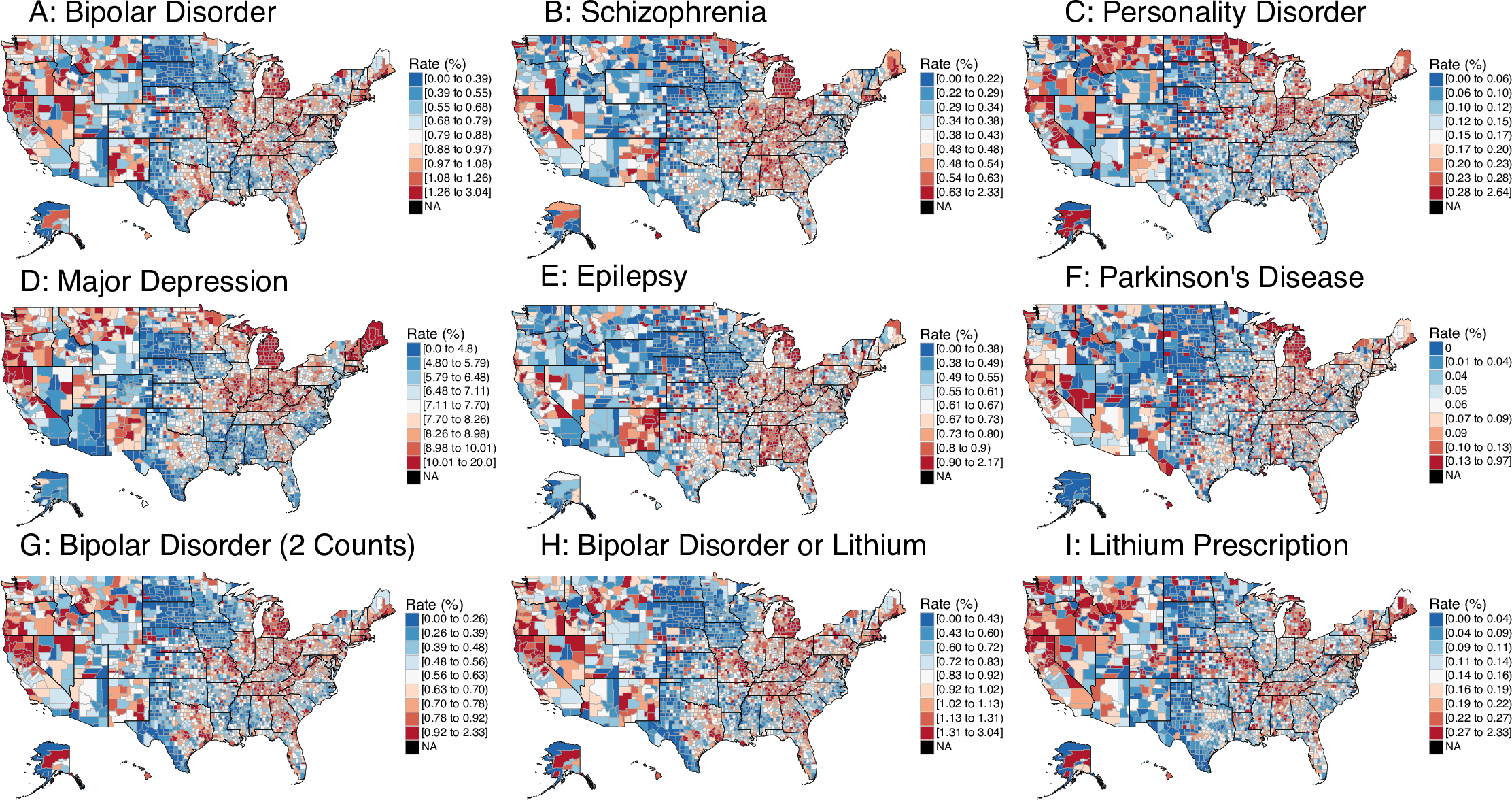

Supplement: S3 Fig — (TIF) [file pbio.3000353.s004.tif]

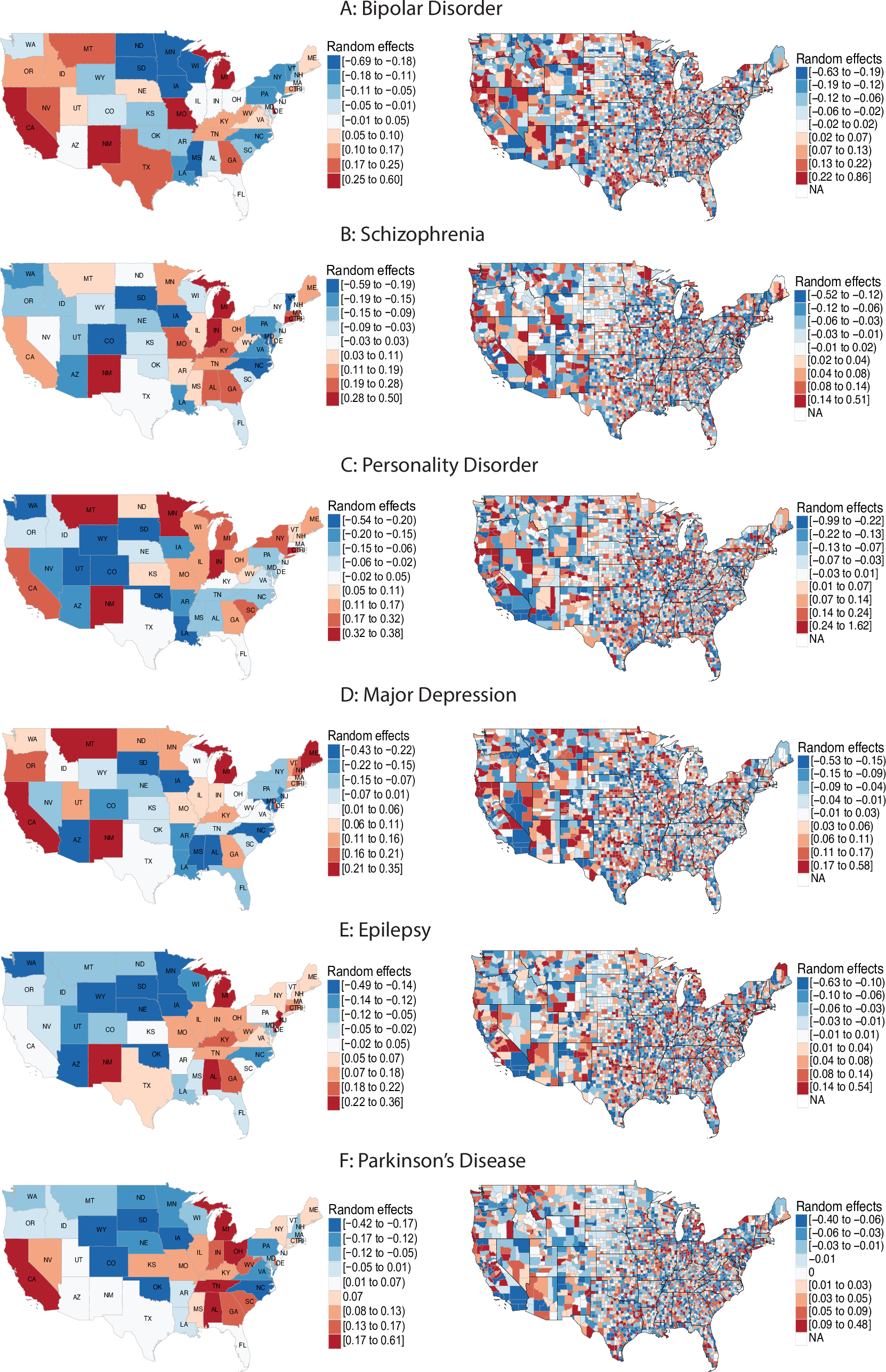

Supplement: S4 Fig — (TIF) [file pbio.3000353.s005.tif]

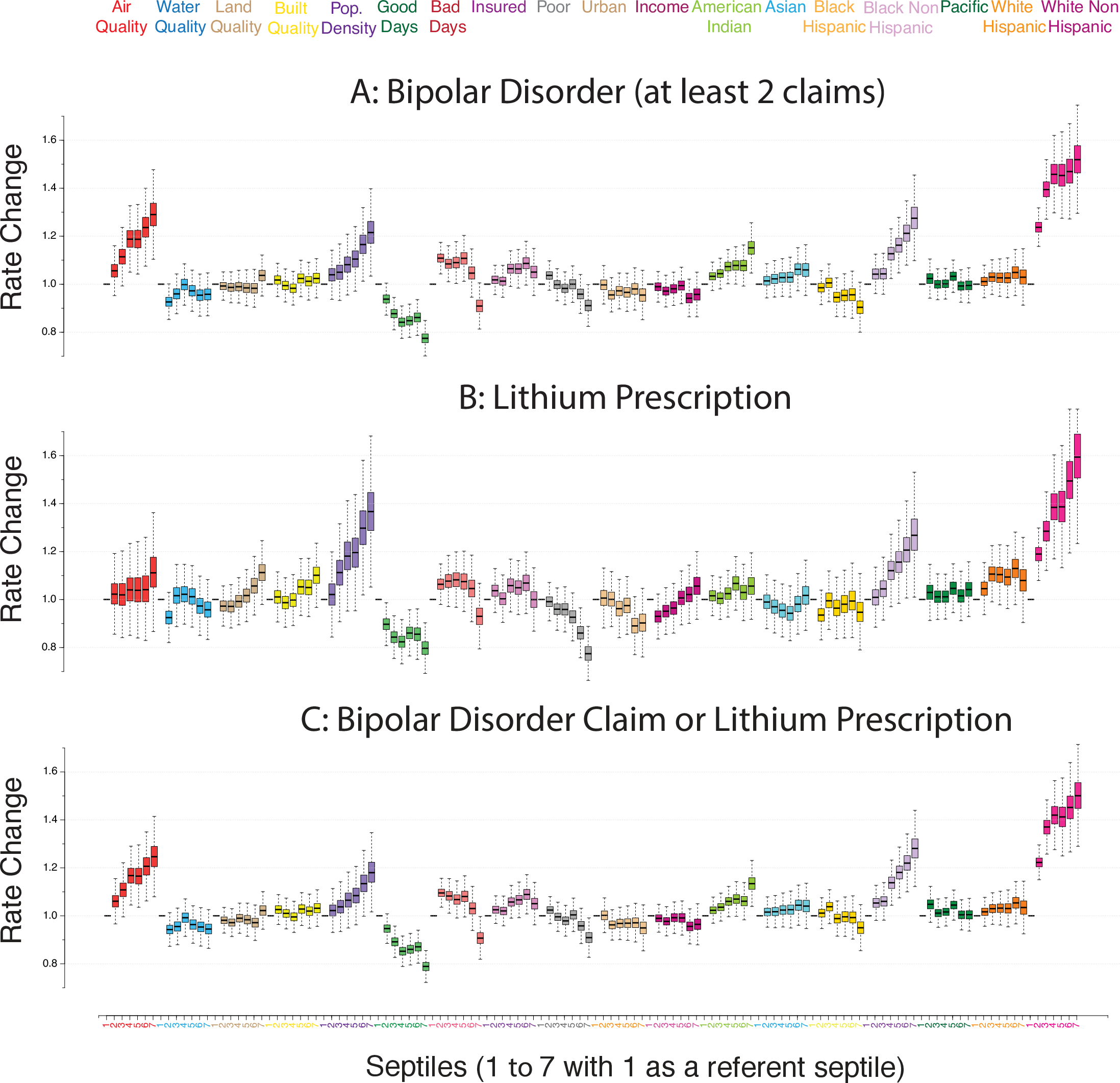

Supplement: S5 Fig — (TIF) [file pbio.3000353.s006.tif]

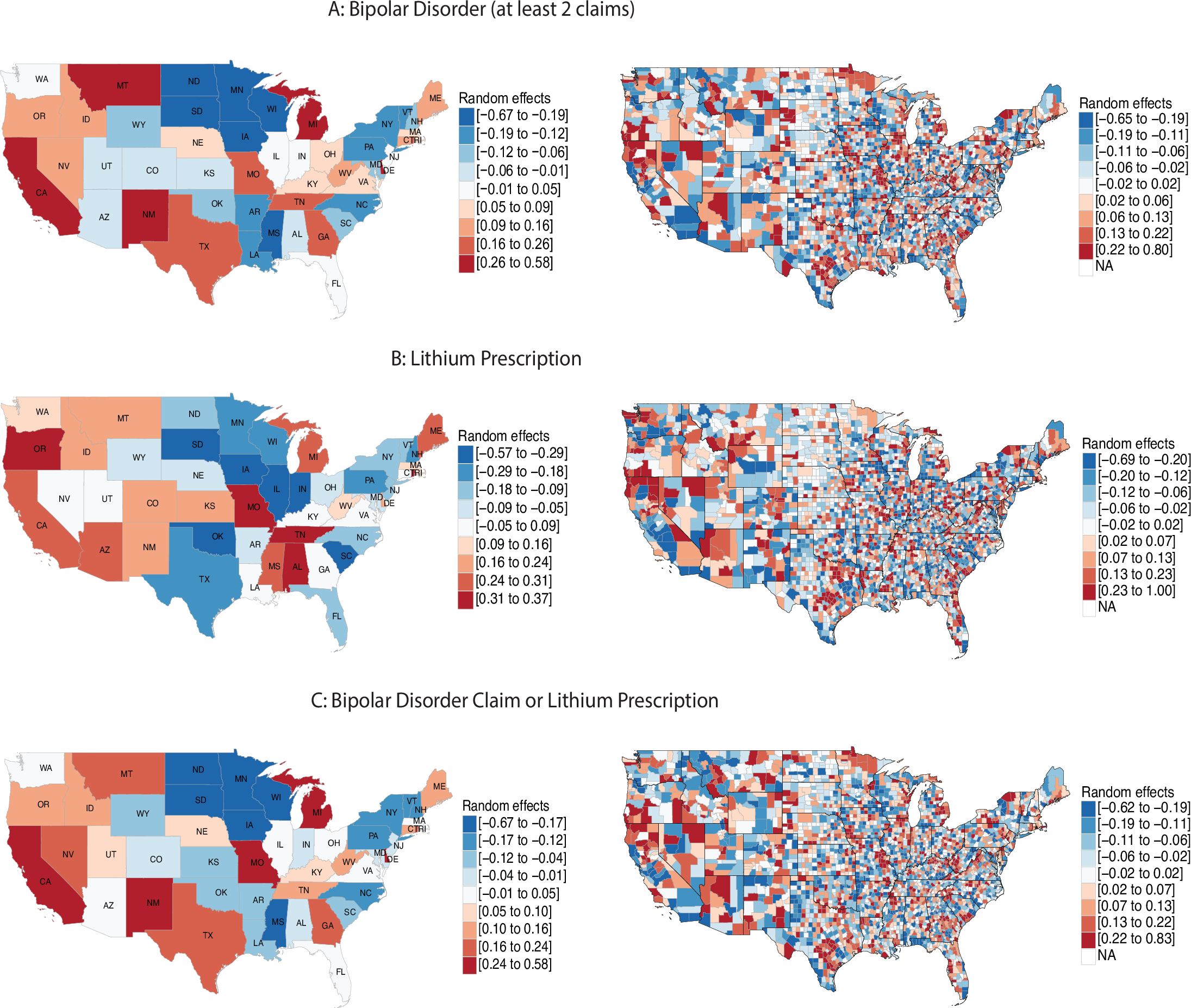

Supplement: S6 Fig — (TIF) [file pbio.3000353.s007.tif]

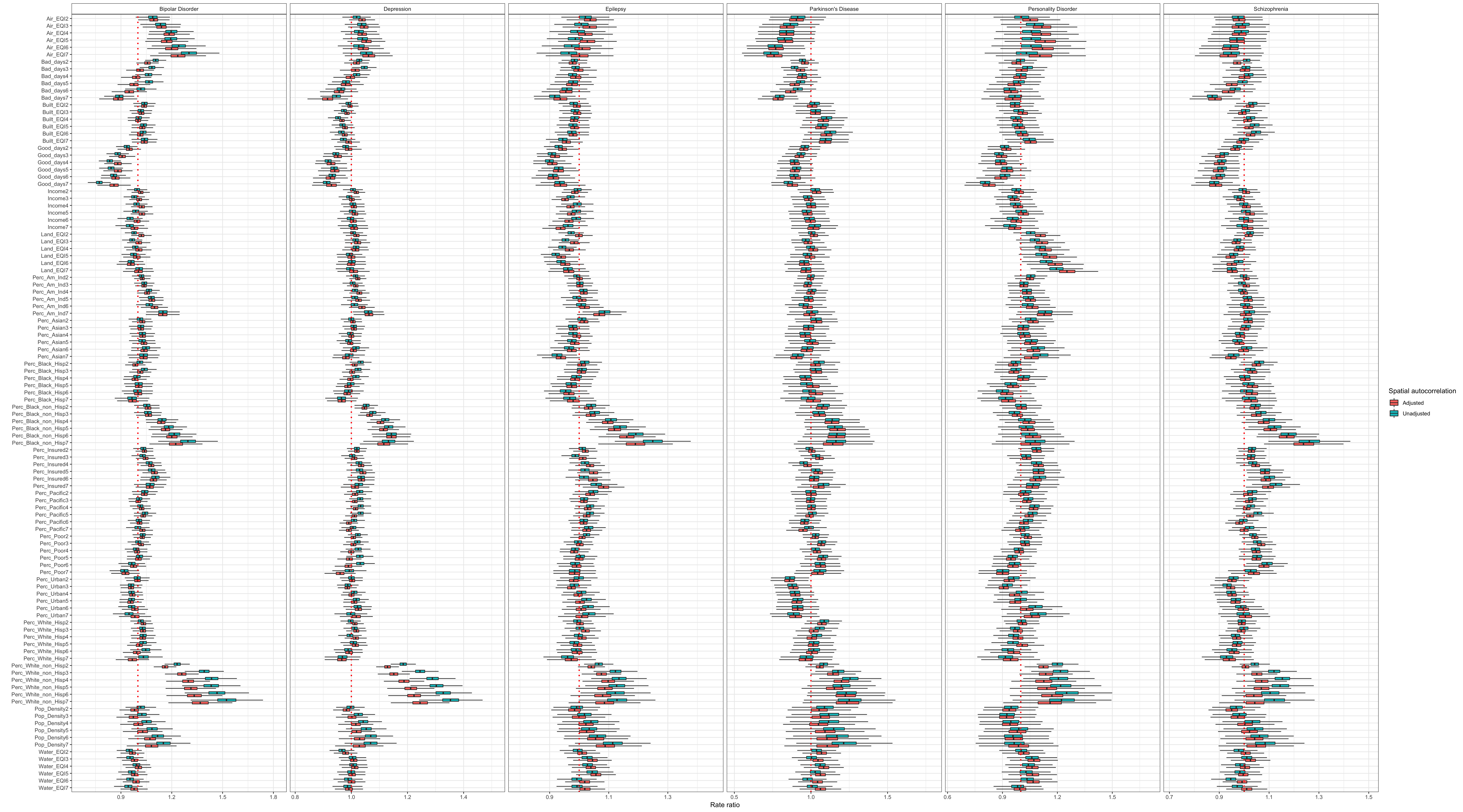

Supplement: S7 Fig — (TIF) [file pbio.3000353.s008.tif]

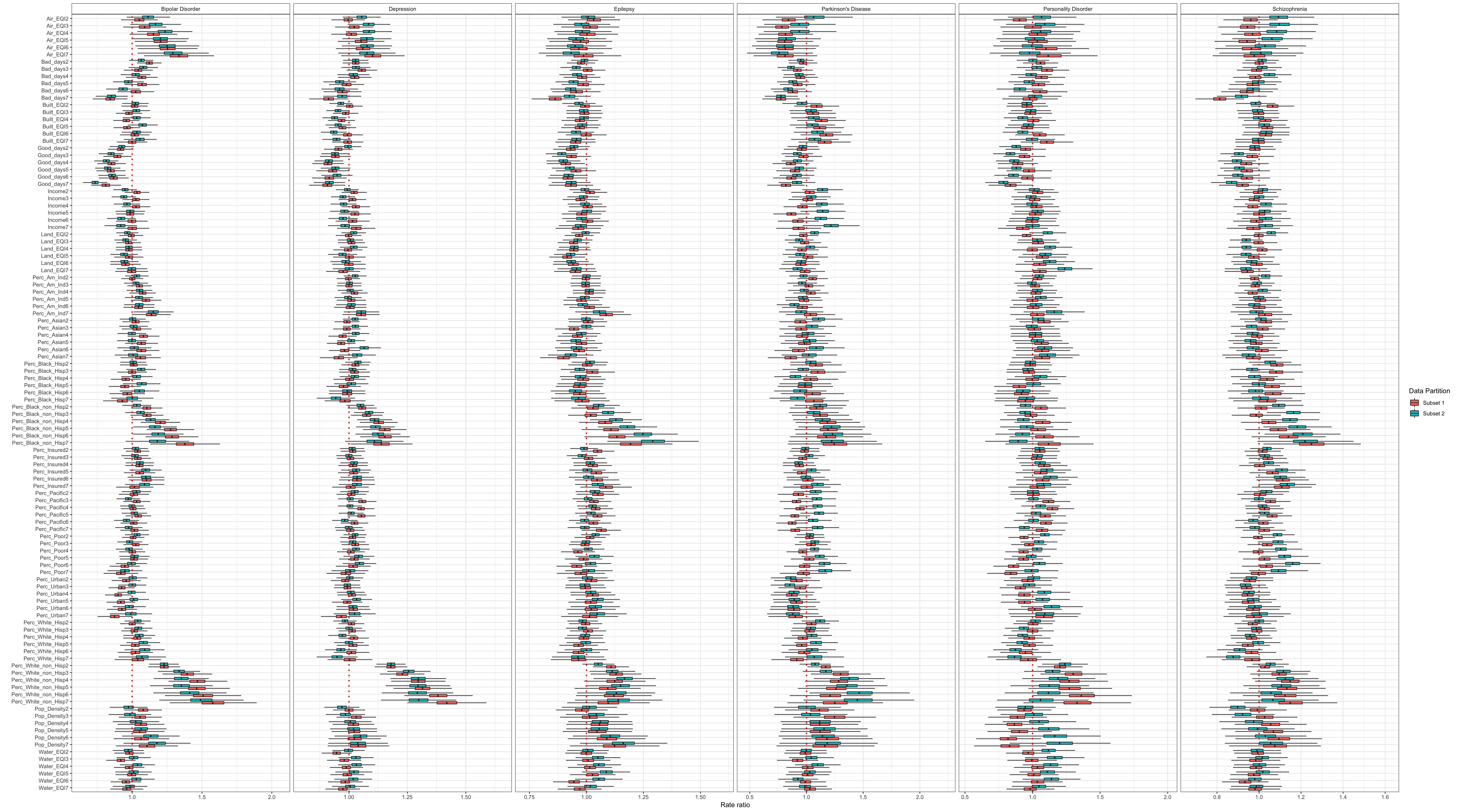

Supplement: S8 Fig — (TIF) [file pbio.3000353.s009.tif]

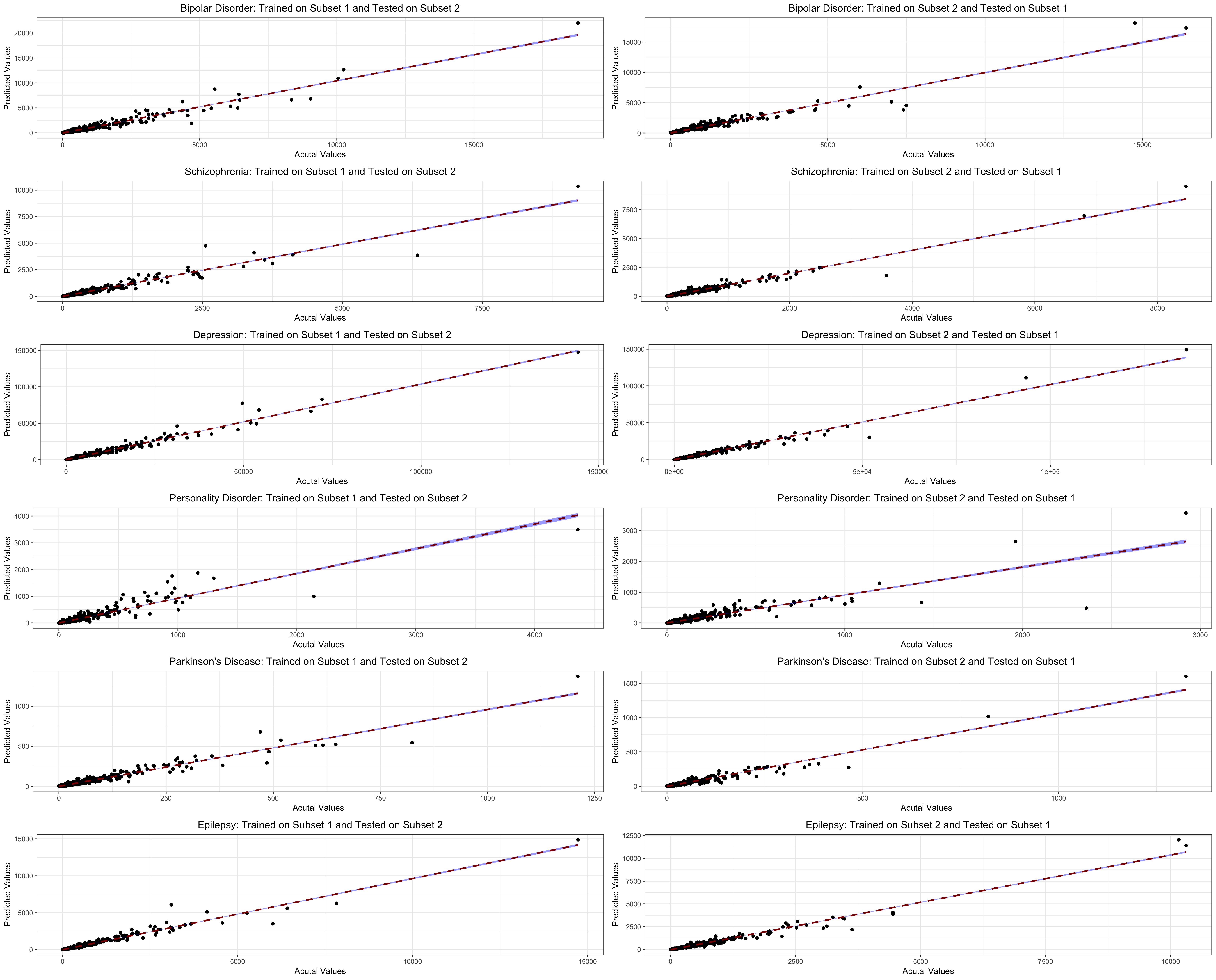

Supplement: S9 Fig — (TIF) [file pbio.3000353.s010.tif]

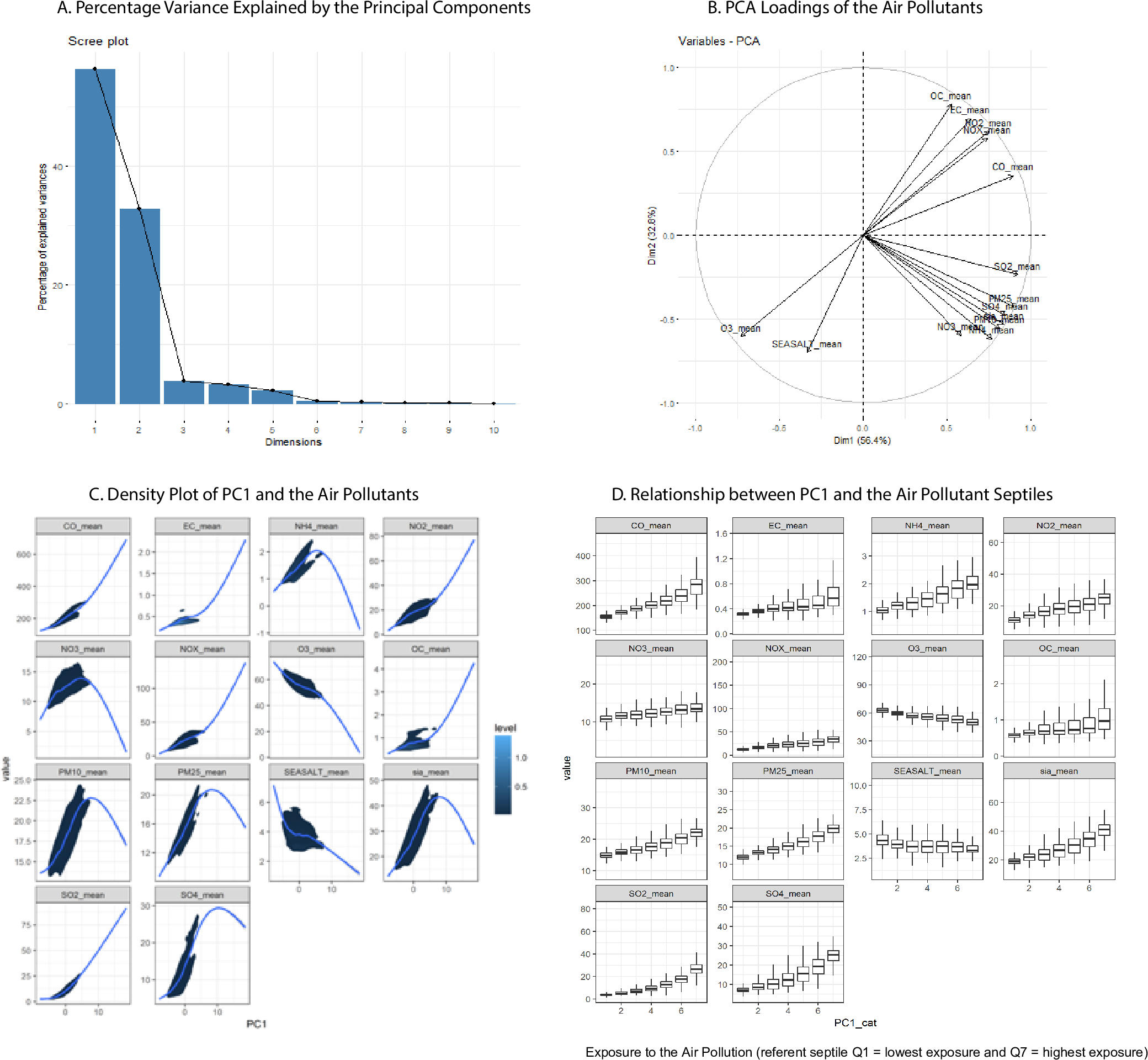

Supplement: S10 Fig — (TIF) [file pbio.3000353.s011.tif]

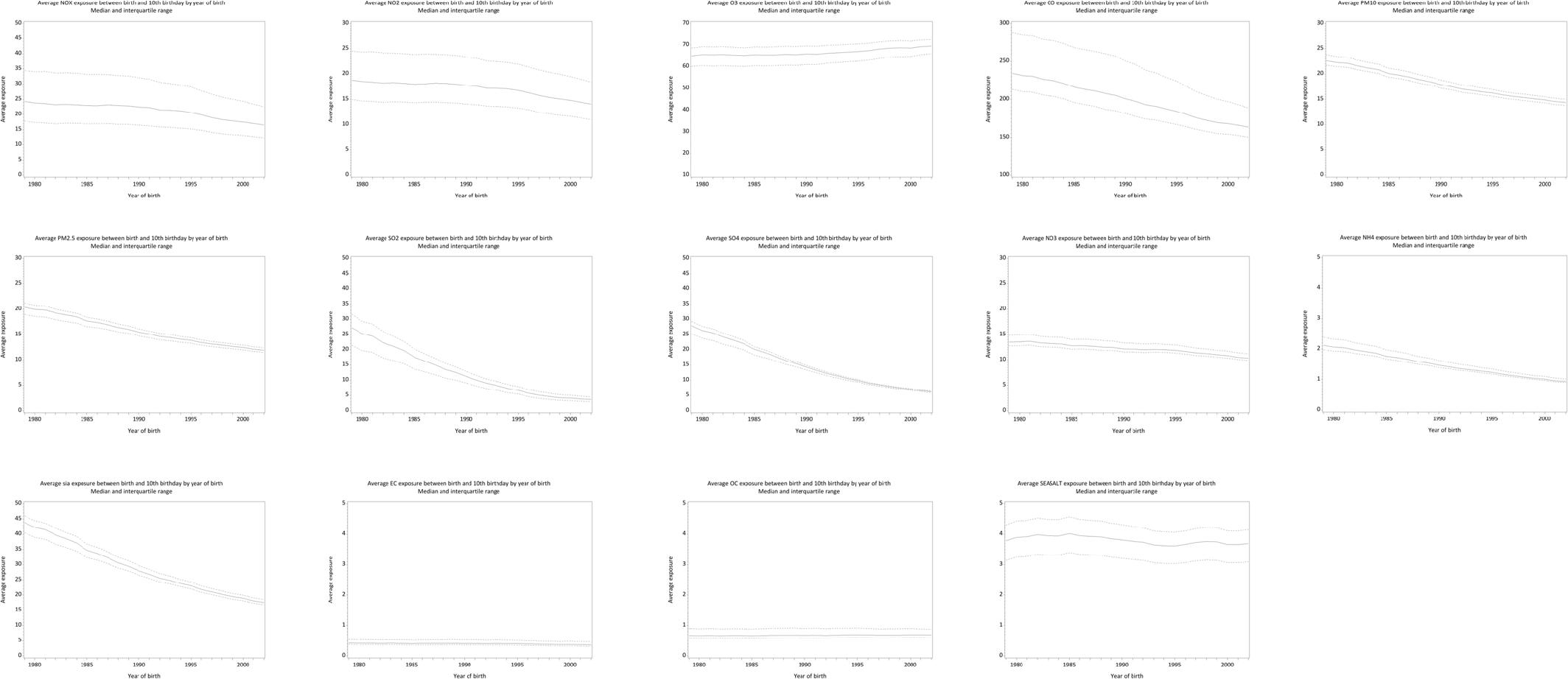

Supplement: S11 Fig — (TIF) [file pbio.3000353.s012.tif]

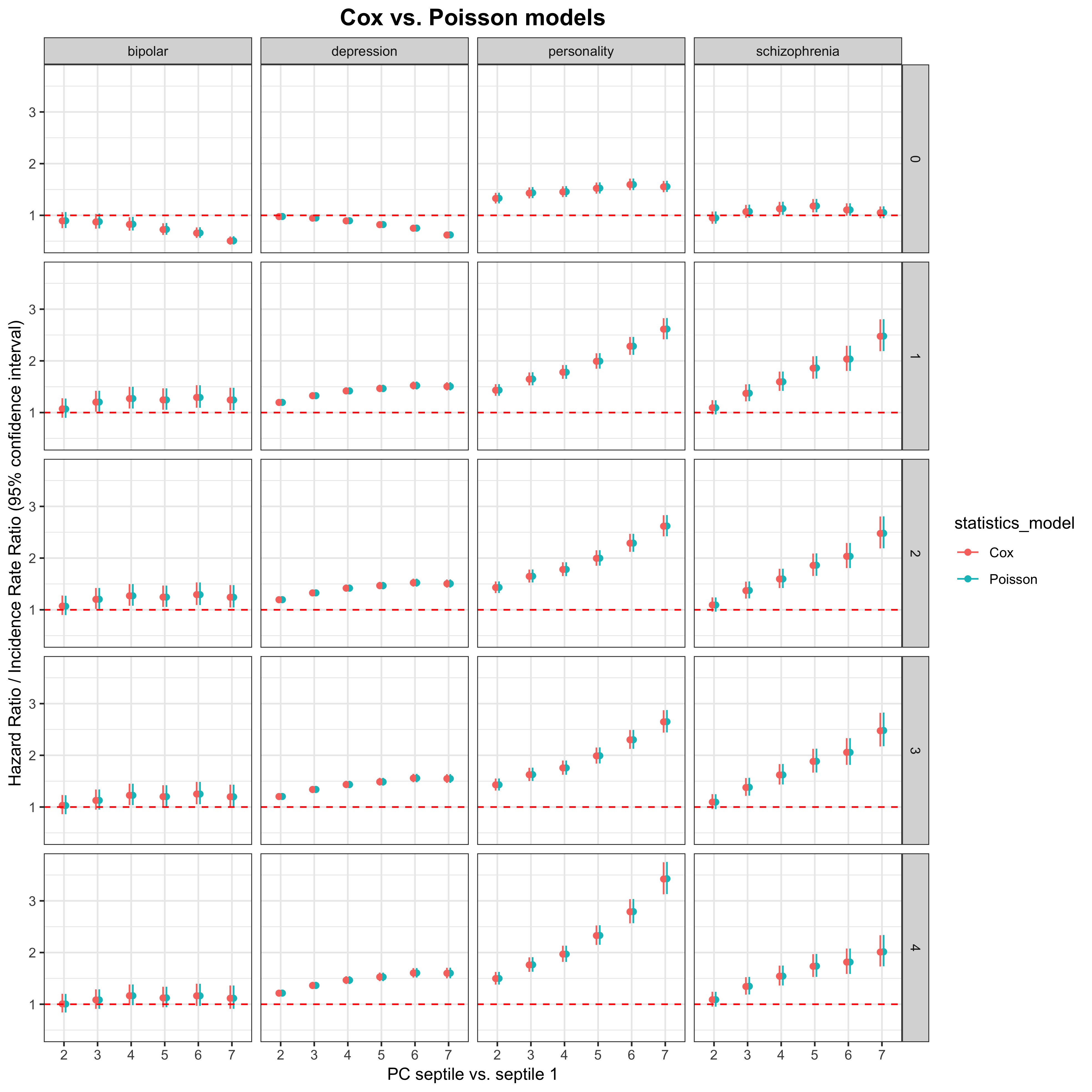

Supplement: S12 Fig — (TIF) [file pbio.3000353.s013.tif]

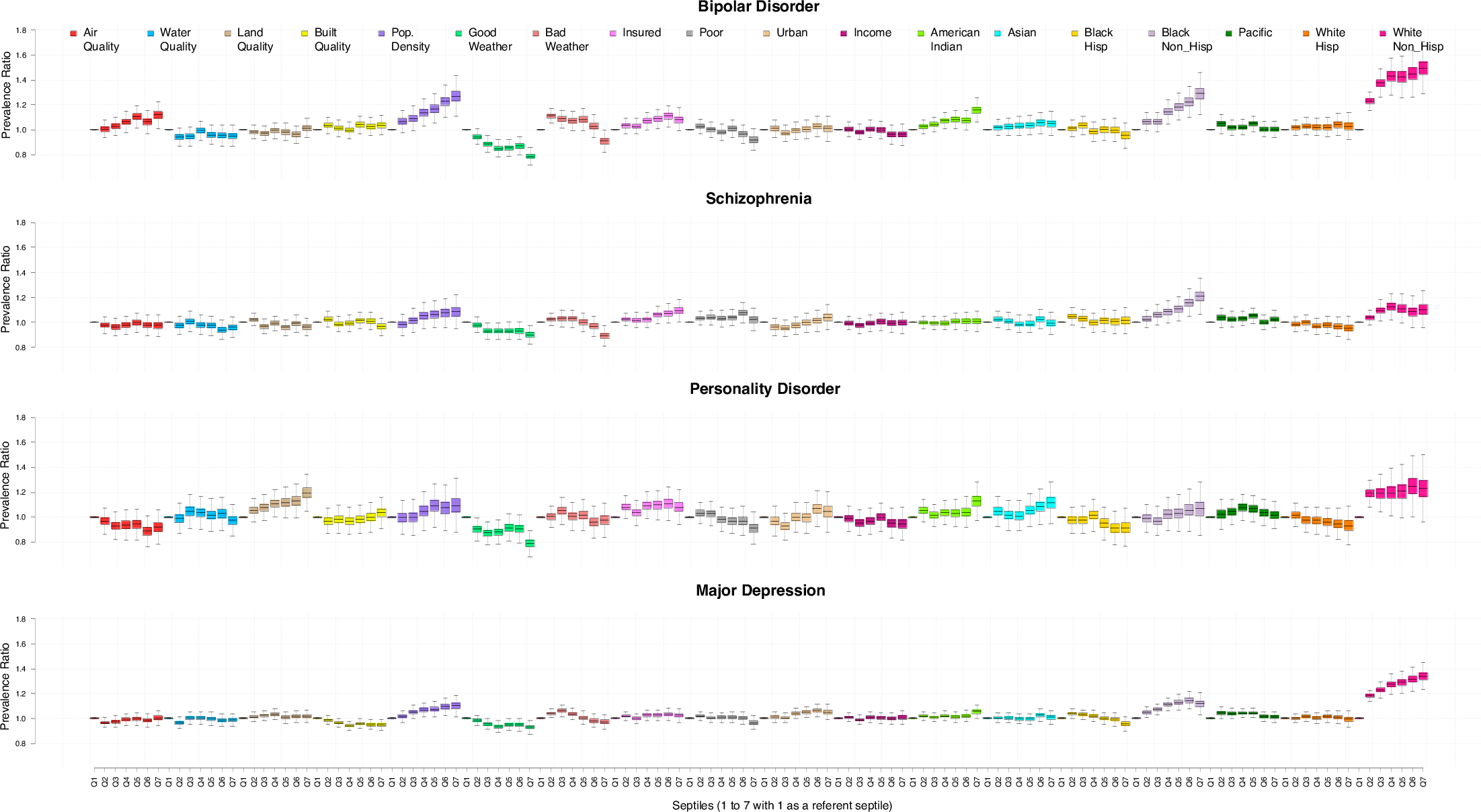

Supplement: S13 Fig — (TIF) [file pbio.3000353.s014.tif]

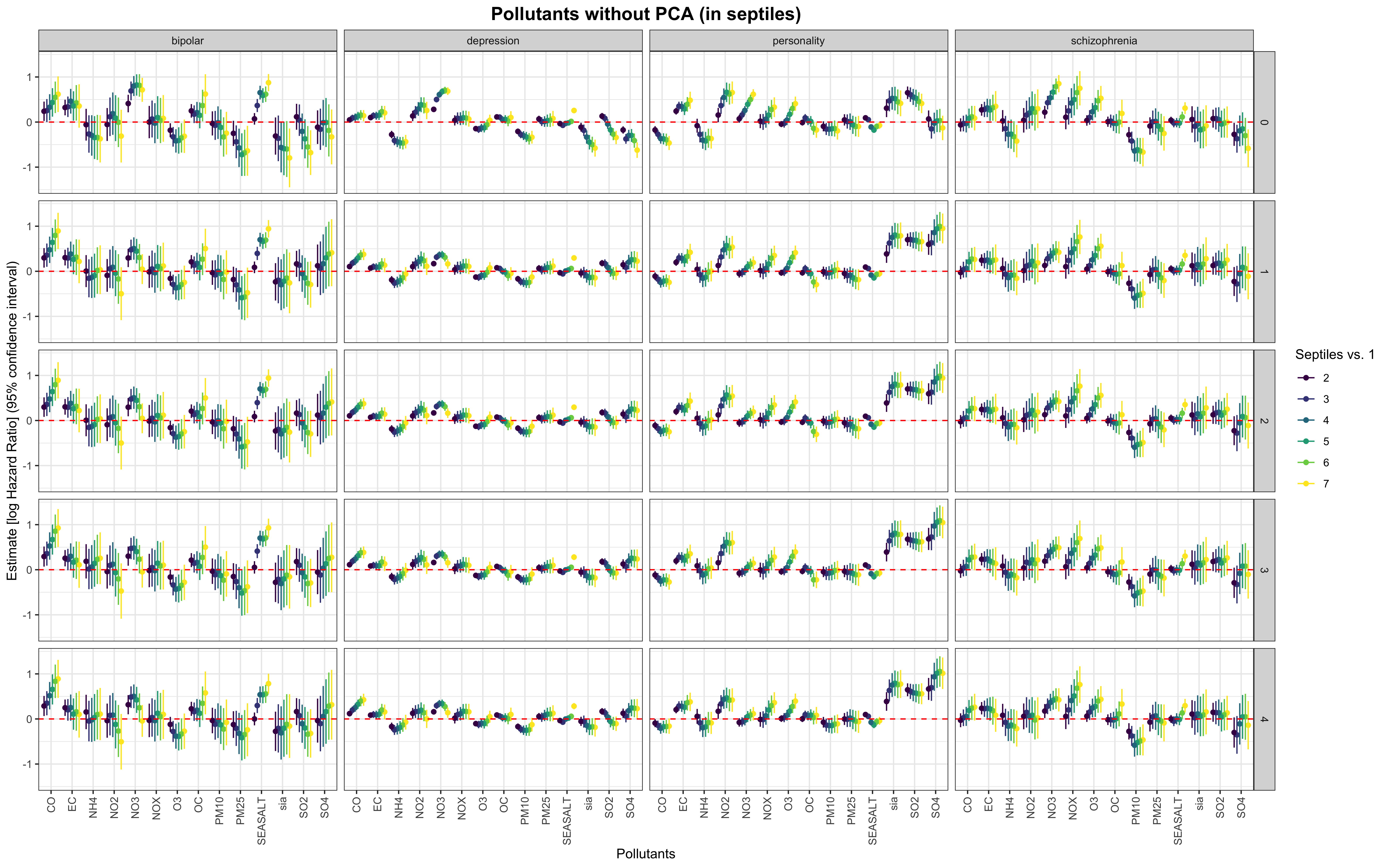

Supplement: S14 Fig — (TIF) [file pbio.3000353.s015.tif]

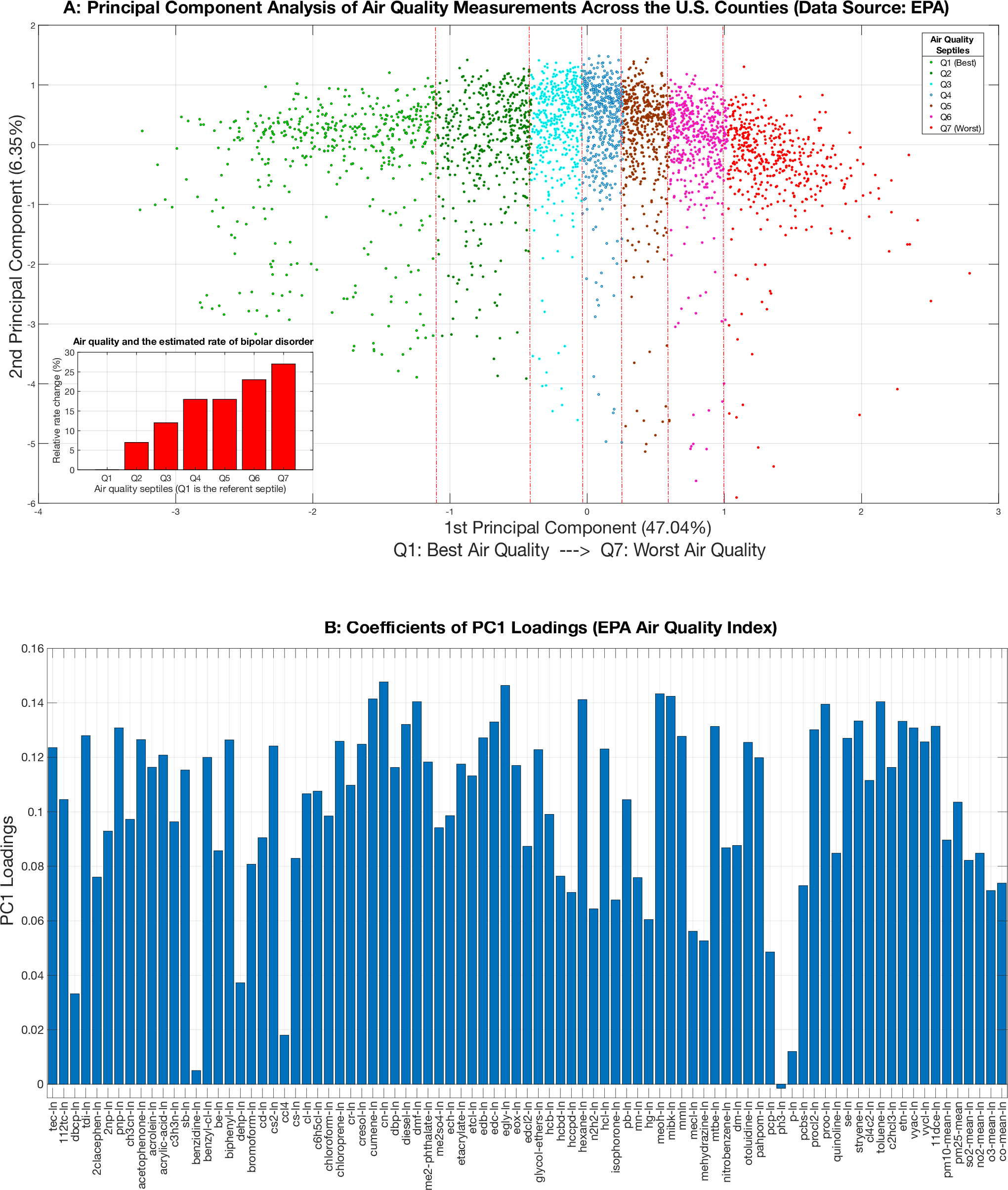

Supplement: S15 Fig — PCA of EPA air quality measurements to produce air quality index. EPA, Environmental Protection Agency; PCA, principal components analysis. (TIF) [file pbio.3000353.s016.tif]
