# Supplementary material for: Environmental pollution is associated with increased risk of psychiatric disorders in the US and Denmark
Source: PLoS Biol. 2019 Aug 20;17(8):e3000353. doi: 10.1371/journal.pbio.3000353 (PMC6701746; doi:10.1371/journal.pbio.3000353)
Supplement: S1 Table — (DOCX) [file pbio.3000353.s017.docx]

#### S1 Table. The estimated prevalence of bipolar disorder, schizophrenia, Parkinson’s disease, personality disorder, epilepsy, and major depression from various published resources.

| **Disease Phenotypes** | **Estimated Prevalence** | **Disease Burden in Millions (Worldwide, US)** | **Gender Effects** | **Source** |
| --- | --- | --- | --- | --- |
| Bipolar Disorder | 1% (General)  2.4-2.6% (Adults) | 60, 5.7 | Higher in Females | NIMH*, WHO[1], CBHSQ[2], Grande et al.[3], Merikangas et al.[4], DiFlorio et al[5]. |
| Schizophrenia | 0.3-0.7% (General)  0.5-1.0% (Adults) | 24, 3.5 | Higher in Males | NIMH*, WHO[1], McGrath et al.[6], Saha et al[6,7]. |
| Parkinson's Disease | 0.1-0.3% (General)  1% (Above 60) | 10, 1 | Higher in Males | NIMH*, Dorsey et al.[8], Nussbaum et al.[9], Tysnes et al.[10], Pringsheim et al.[11] |
| Personality Disorder | 1-2% (General)  5.9% (Adults) | NA, 14 | Higher in Females | NIMH*, NAMI+, Lenzenweger et al.[12], Zanarini et al.[13] |
| Epilepsy | 0.7-1.2% (General)  0.6% (Under 18) | 50, 3.4 | Almost the Same | CDC**-**, WHO[14], Zack et al.[15], Fiest et al.[16], Russ et al[17]. |
| Major Depression | 3-5.5% (General)  5.3-6.7% (Adults) | 300, 16 | Higher in Females | NIMH, WHO[18], CBHSQ[2], Ferrari et al.[19], Kessler et al.[20] |

* National Institute of Mental Health: <https://www.nimh.nih.gov/index.shtml>

+ National Alliance on Mental Health: <https://www.nami.org>

**-** Centers for Disease Control and Prevention (CDC): <https://www.cdc.gov>

# References

1. World Health Organisation. The World Health Report 2001: mental health, new understanding, new hope. In: World Health Report [Internet]. 2001 pp. 1–169. Available: http://www.who.int/whr/2001/en/whr01_en.pdf

2. SAMHSA C. Results from the 2015 National Survey on Drug Use and Health: Detailed Tables,. 2015 Natl Surv Drug Use Heal. 2015; 209. Available: https://www.samhsa.gov/data/sites/default/files/NSDUH-DetTabs-2015/NSDUH-DetTabs-2015/NSDUH-DetTabs-2015.htm#tab5-1c

3. Grande I, Berk M, Birmaher B, Vieta E. Bipolar disorder. The Lancet. 2016. pp. 1561–1572. doi:10.1016/S0140-6736(15)00241-X

4. Merikangas KR, Jin R, He J-P, Kessler RC, Lee S, Sampson NA, et al. Prevalence and correlates of bipolar spectrum disorder in the world mental health survey initiative. Arch Gen Psychiatry. 2011;68: 241–51. doi:10.1001/archgenpsychiatry.2011.12

5. DiFlorio A, Jones I. Is sex important? Gender differences in bipolar disorder. Int Rev Psychiatry. 2010;22: 437–52. doi:10.3109/09540261.2010.514601

6. Saha S, Chant D, Welham J, McGrath J. A systematic review of the prevalence of schizophrenia. PLoS Medicine. 2005. pp. 0413–0433. doi:10.1371/journal.pmed.0020141

7. McGrath J, Saha S, Chant D, Welham J. Schizophrenia: A concise overview of incidence, prevalence, and mortality. Epidemiologic Reviews. 2008. pp. 67–76. doi:10.1093/epirev/mxn001

8. Dorsey ER, Constantinescu R, Thompson JP, Biglan KM, Holloway RG, Kieburtz K, et al. Projected number of people with Parkinson disease in the most populous nations, 2005 through 2030. Neurology. 2007. pp. 384–386. doi:10.1212/01.wnl.0000247740.47667.03

9. Nussbaum RL, Ellis CE. Alzheimer’s Disease and Parkinson’s Disease. N Engl J Med. 2003; 1356–64. doi:10.1056/NEJM2003ra020003

10. Tysnes OB, Storstein A. Epidemiology of Parkinson’s disease. Journal of Neural Transmission. 2017. pp. 901–905. doi:10.1007/s00702-017-1686-y

11. Pringsheim T, Jette N, Frolkis A, Steeves TDL. The prevalence of Parkinson’s disease: A systematic review and meta-analysis. Movement Disorders. 2014. pp. 1583–1590. doi:10.1002/mds.25945

12. Lenzenweger MF, Lane MC, Loranger AW, Kessler RC. DSM-IV Personality Disorders in the National Comorbidity Survey Replication. Biol Psychiatry. 2007;62: 553–564. doi:10.1016/j.biopsych.2006.09.019

13. Zanarini MC, Horwood J, Wolke D, Waylen A, Fitzmaurice G, Grant BF. Prevalence of DSM-IV Borderline Personality Disorder in Two Community Samples: 6,330 English 11-Year-Olds and 34,653 American Adults. J Pers Disord. 2011;25: 607–619. doi:10.1521/pedi.2011.25.5.607

14. Kessler RC, Aguilar-Gaxiola S, Alonso J, Chatterji S, Lee S, Ormel J, et al. The global burden of mental disorders: an update from the WHO World Mental Health (WMH) surveys. Epidemiol Psichiatr Soc. 2009;18: 23–33. doi:10.1017/S1121189X00001421

15. Zack MM, Kobau R. National and State Estimates of the Numbers of Adults and Children with Active Epilepsy — United States, 2015. MMWR Morb Mortal Wkly Rep. 2017;66: 821–825. doi:10.15585/mmwr.mm6631a1

16. Fiest KM, Sauro K, Wiebe S, Patten S, Kwon C, Dykeman J, et al. The prevalence and incidence of epilepsy: A systematic review and meta-analysis of international studies. Neurology. 2017; doi:10.1212/WNL.0000000000003509

17. Russ SA, Larson K, Halfon N. A National Profile of Childhood Epilepsy and Seizure Disorder. Pediatrics. 2012;129: 256–264. doi:10.1542/peds.2010-1371

18. World Health Organization. Depression and other common mental disorders: global health estimates. World Heal Organ. 2017; 1–24. doi:CC BY-NC-SA 3.0 IGO

19. Ferrari a. J, Somerville a. J, Baxter a. J, Norman R, Patten SB, Vos T, et al. Global variation in the prevalence and incidence of major depressive disorder: a systematic review of the epidemiological literature. Psychol Med. 2012; 1–11. doi:10.1017/S0033291712001511

20. Kessler RC, Bromet EJ. The Epidemiology of Depression Across Cultures. Annu Rev Public Health. 2013;34: 119–138. doi:10.1146/annurev-publhealth-031912-114409
